# Endogenous formation of phosphatidylhomoserine in *Escherichia coli* through phosphatidylserine synthase

**DOI:** 10.1016/j.jbc.2025.110255

**Published:** 2025-05-20

**Authors:** Elise Zi Qi Ng, Eunju Lee, Shu-Sin Chng, Jungwook Kim, Xue Li Guan

**Affiliations:** 1 Lee Kong Chian School of Medicine, Nanyang Technological University, Singapore; 2 Department of Chemistry, Gwangju Institute of Science and Technology, Gwangju, Republic of Korea; 3 National University of Singapore, Department of Chemistry, Singapore

**Keywords:** Gram-negative bacteria, phospholipid metabolism, enzyme, substrate specificity, mass spectrometry, phosphatidylserine synthase, homoserine, lipidome

## Abstract

Biological membranes, which comprise proteins, lipids, and glycans, serve as essential gatekeepers protecting cells from the external environment. In bacteria, phospholipids are a major class of membrane lipids, whose biology has extensively been studied in the Gram-negative organism *Escherichia coli*. As an adaptive mechanism, *E. coli* dynamically remodels its phospholipids in response to its environment, which may involve alterations of the structures and/or levels of existing lipids or the incorporation of exogenous substrates to form new phospholipid classes. Intriguingly, an unknown lipid was detected in *E. coli* and other Enterobacteriaceae. Detection of this lipid in *E. coli* grown in minimal media suggested its production using an endogenous metabolite. By coupling liquid chromatography mass spectrometry and metabolic incorporation, the lipid was identified as phosphatidylhomoserine (PHS). In *E. coli*, PHS was produced endogenously by phosphatidylserine synthase A (PssA), confirmed by the absence of PHS in an *E. coli* Δ*pssA* mutant, and its inability to incorporate exogenously supplied _L_-homoserine into its phospholipids. Furthermore, purified *E. coli* PssA (*Ec*PssA) exhibited activity to utilize _L_-homoserine as an alternative substrate to make PHS *in vitro*. Interestingly, *E. coli* and other Enterobacteriaceae can decarboxylate PHS to form phosphatidylpropanolamine endogenously. When treated with _L_-homoserine, accumulation of PHS in *E. coli* was accompanied by a reduction in phosphatidylglycerol and phosphatidylethanolamine, due to competition for common metabolic intermediates. Overall, our findings on the endogenous production of PHS and phosphatidylpropanolamine re-established the baseline phospholipidome of *E. coli* and provided biochemical and cellular evidence on the substrate promiscuity of *Ec*PssA.

Phospholipids, besides lipopolysaccharides, glycans, and proteins, are the major components of Gram-negative bacterial membranes which form a protective barrier from the extracellular environment (1) and play important roles in diverse biological processes (2, 3, 4). As a unicellular organism, bacteria have evolved strategies to cope with its varying environment, and phospholipid remodeling presents one of the key mechanisms for rapid survival responses to perturbations (5, 6). Studies on the model organism, *Escherichia coli*, have laid a strong foundation for our current understanding of phospholipid metabolism, transport, and functions in Gram-negative bacteria (3, 4, 7, 8, 9, 10, 11).

As a diderm, *E. coli* has two membrane bilayers, which comprise of an outer membrane enriched in lipopolysaccharides, while phospholipids form the main lipid component of the inner membrane. Compositionally, phosphatidylethanolamine (PE) accounts for up to 70% of the total phospholipid content in *E. coli*, followed by phosphatidylglycerol (PG), cardiolipin (CL), and phosphatidic acid (PA) (7). Phospholipid synthesis in *E. coli* starts with the sequential acylation of glycerol-3-phosphate by acyltransferases, PlsB and PlsC to form PA (12, 13, 14). Cytidine diphosphate diacylglycerol (CDP-DAG), which is next formed from PA by CDP-DAG synthase, CdsA (15, 16), is the metabolic branch point for the formation of the two major diacyl phospholipids, PE and PG (Fig. 1*A*) (11, 17). The primary pathway for PE synthesis in *E. coli* is through phosphatidylserine (PS) decarboxylation (11). PS is produced through the transfer of serine to CDP-DAG by phosphatidylserine synthase, PssA (18, 19), followed by decarboxylation to yield PE through the actions of phosphatidylserine decarboxylase, Psd (20, 21, 22). PG, on the other hand, is formed from the metabolic intermediate, phosphatidylglycerol phosphate (PGP), synthesized by phosphatidylglycerol synthase, PgsA (23). PGP is in turn dephosphorylated to form PG and subsequently CL through the actions of PGP phosphatases, PgpA/B/C, and cardiolipin synthases, ClsA/B/C respectively (23, 24, 25, 26). Besides their main activities, it is unsurprising that enzymes can exhibit additional activities, either due to the presence of multiple functional domains or substrate promiscuity (27). For instance, PssA is able to incorporate phosphoglycerol, which ultimately leads to the formation of PG, albeit at low level (28). In *E. coli*, nonclassical phospholipids, phosphatidylalcohols, can be formed with the introduction of exogenous primary alcohols (29), through the phospholipase D (PLD) activity of ClsB (30). When cultured in the presence of mannitol, *E. coli* was also able to form two novel lipids, phosphatidylmannitol and diphosphatidylmannitol, contributed by the activity of cardiolipin synthase (31).

**Figure 1:**
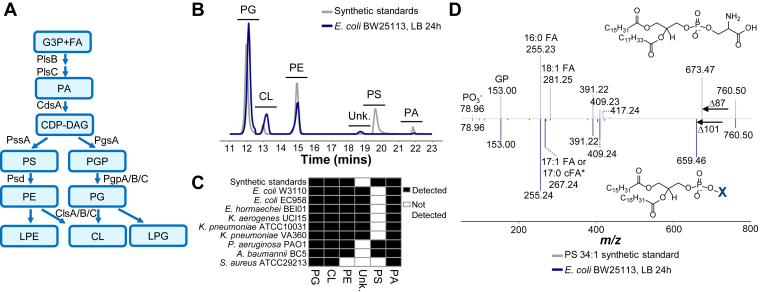
***Escherichia coli* phospholipid pathway and discovery of a previously uncharacterized lipid.***A*, general phospholipid pathway of *E. coli*. *B*, normal phase chromatography separation of *E. coli* phospholipid fraction (*blue*) and phospholipid synthetic standards (*gray*), for classical phospholipid classes, PE, PG, CL, PA, PS, found in Gram-negative bacteria. Peaks represent extracted ion chromatogram (XIC) of the main or major ions of each lipid class. An unknown peak in *E. coli* eluting close to (but before) PS 34:1 synthetic standard was detected. *C*, conservation of the unknown lipid in Enterobacteriaceae species. Lipids were isolated from various bacterial species and analyzed using LC/MS^2^. The heatmap represents the absence (*white*) or presence (*black*) of a phospholipid class, based on the analytical detection in this study. *D*, MS^2^ spectra of PS 34:1 (16:0/18:1) synthetic standard (*top* spectrum, *gray*) and the unknown ion detected in *E. coli* (*bottom*, inverted spectrum, *blue*). Fragmentation of PS-generated diagnostic ions from the neutral loss of serine (87 Da), phosphate (*m/z* 78.96), glycerophosphate and fatty acids, 18:1 (*m/z* 281.25) and 16:0 (*m/z* 255.23). For the ion found in *E. coli*, it exhibited a neutral loss of 101 Da. Other diagnostic ions include the signature of phosphate, glycerophosphate, and fatty acids, 16:0 (*m/z* 255.24) and 17:1 or c17:0 (*m/z* 267.24). Inset showed the structures of PS and partial structure of the unknown, with its headgroup ‘X’ to be further characterized. MS^2^ data was derived from one sample and is representative of the three biological replicates. CDP-DAG, cytidine diphosphate diacylglycerol; CdsA, phosphatidate cytidylyltransferase; cFA, cyclopropane fatty acid; CL, cardiolipin; ClsA/B/C, cardiolipin synthase A/B/C; Da, Dalton; FA, fatty acid; G3P, glycerol-3-phosphate; GP, glycerophosphate; LB, Luria-Bertani broth (Miller); LC/MS^2^, liquid chromatography-high resolution tandem mass spectrometry; LPE, lysophosphatidylethanolamine; *m/z*, mass-to-charge ratio; PA, phosphatidic acid; PE, phosphatidylethanolamine; PG, phosphatidylglycerol; PGP, phosphatidylglycerol phosphate; PgpA/B/C, phosphatidylglycerophosphatase A/B/C; PgsA, phosphatidylglycerol phosphate (PGP) synthase; PlsB, glycerol-3-phosphate acyltransferase; PlsC, 1-acyl-sn-glycerol-3-phosphate acyltransferase; PO3, phosphate; PS, phosphatidylserine; Psd, phosphatidylserine decarboxylase; PssA, phosphatidylserine synthase; Unk., unknown. ∗The analytical method does not distinguish between mono-unsaturated fatty acid and cyclopropane fatty acid.

The lipid composition of a unicellular bacterium is clearly shaped by the combination of its enzymatic machinery as well as substrate availability. *In vitro* substrate characterization with purified enzymes are fundamental and powerful techniques which have contributed significantly to our current understanding of the specific activities of lipid enzymes in diverse organisms. However, biological systems are inherently complex, and both intracellular and extracellular conditions can influence enzymatic activities, ultimately changing the biochemical profile of a bacterium (29). In addition, key lipid precursors such as serine and acetate intersect with pathways involved in protein synthesis and carbon metabolism (32). As substrates are funneled into different biochemical pathways, the lipid profile of a cell is therefore dependent on both the environmental conditions and its physiological state. Liquid chromatograph mass spectrometry (LC/MS) offers unprecedented resolution and sensitivity to probe the biochemistry and metabolic state of a cell. Indeed, whole cell lipidome analysis is a complementary approach to understand global cellular biochemistry (33, 34). This systems-scale approach had been applied by various groups to study lipid metabolism and functions in bacteria, and PE, PG, and CL had been coherently reported as the major phospholipids found in *E. coli* (29, 35, 36). However, there are existing variations in other components detected, which can arise from the bioanalytical methods, bacterial strains, and culture conditions used. For instance, the presence of phosphatidylserylglutamate (37), phosphatidylmannitol (31), and phosphatidylalcohols (30, 38) had been described in *E. coli*. Nevertheless, not all nonclassical phospholipids can be attributed to the existing biochemical machinery found in *E. coli*, and further works will be required to confirm their true existence and biological functions.

In this study, we described the characterization of two novel endogenous phospholipids present in *E. coli* and other Enterobacteriaceae. Using liquid chromatography-high resolution tandem mass spectrometry (LC/MS^2^), substrate incorporation, genetics, and enzyme biochemistry, we provided structural and biochemical evidence of phosphatidylhomoserine (PHS) in *E. coli*. PHS is formed endogenously through the activity of *Ec*PssA. The ability of *Ec*PssA to use _L_-homoserine (Hse) as a substrate to form PHS was demonstrated in cells, as well as using purified enzyme system *in vitro*. *E. coli* can further decarboxylate PHS to form phosphatidylpropanolamine (PPA) endogenously, albeit at very low levels. When exposed to _L_-homoserine (Hse), the bacterium accumulated PHS and PPA, with reduction in PG and PE levels potentially due to the competition of the common building block, CDP-DAG. This work renewed our fundamental understanding of *E. coli* lipid metabolism at the cellular level; specifically, our findings re-established the baseline phospholipidome of *E. coli* and revealed the previously uncharacterized substrates of *Ec*PssA. Furthermore, our works also demonstrated the type of substrates and degree of incorporation into phospholipids depend on the bacterial species and the type of growth media, highlighting environmentally driven plasticity and complexity of bacterial lipid metabolism.

## Results

### Baseline phospholipidome of *E. coli* harbors an unknown, which is conserved in Enterobacteriaceae

The phospholipidome of *E. coli* cultured in LB broth (Miller) (LB) was analyzed using normal phase chromatography with tandem mass spectrometry (MS^2^), which separated phospholipids by their headgroups (Fig. 1*B*). Consistent with existing reports (29, 35, 36), PE, PG, and CL are the major phospholipid classes detected in *E. coli* strain BW25113 under aerobic conditions in LB, a standard laboratory media. PA was detected at low levels, and an additional peak was observed, which eluted before but close to PS synthetic standard, with ions sharing the same mass-to-charge ratio (*m/z*) as PS 34:1. This peak was consistently found in three *E. coli* strains (*E. coli* BW25113, *E. coli* W3110, and *E. coli* EC958), as well as other Enterobacteriaceae (*Klebsiella pneumoniae*, *Klebsiella aerogenes*, *Enterobacter. hormaechei*) (Fig. 1*C*). However, it was not detectable in non-Enterobacteriaceae Gram-negative bacteria, *Acinetobacter baumannii* or *Pseudomonas aeruginosa*; instead, PS was detected (Fig. 1*B*). Both the unknown and PS were absent in the Gram-positive bacterium, *Staphylococcus aureus*.

Based on the difference in retention time from PS, we postulated *E. coli* and other Enterobacteriaceae can produce another class of phospholipids. MS^2^ analyses revealed that in contrast to a neutral loss of 87 Da (Da), arising from the loss of serine from PS, the fragmentation of the ion found in *E. coli* formed a neutral loss of 101 Da (Fig. 1*D*). The *E. coli* lipid possessed the signature phosphate (*m/z* 78.96) and glycerophosphate (*m/z* 153.00) fragments, which were also found in the PS fragmentation profile (Fig. 1*D*). The major form of this ion contained two fatty acyls, 16:0 fatty acid and a 17-carbon fatty acid with either one double bond or cyclopropane modification. In the parasite, *Toxoplasma gondii* (39), as well as human blood (40), phosphatidylthreonine (PT), which forms a neutral loss of 101 Da during fragmentation, had been previously described (40). Indeed, threonine is 14 Da larger than serine, and intuitively as one of the essential amino acids produced in the cells, it should be a readily available substrate. PT in *T. gondii* is synthesized by PT synthase (*Tg*PTS) (39); however, no homolog of *Tg*PTS is found in *E. coli*, based on BLAST sequence alignment query. One possibility would be to form PT in *E. coli* through the activity of PssA. However, Kanfer and Kennedy demonstrated that PssA does not harbor observable activity when threonine was used as a substrate (11).

### Identification of PHS in *E. coli* through substrate incorporation in whole bacterium

**Figure 2:**
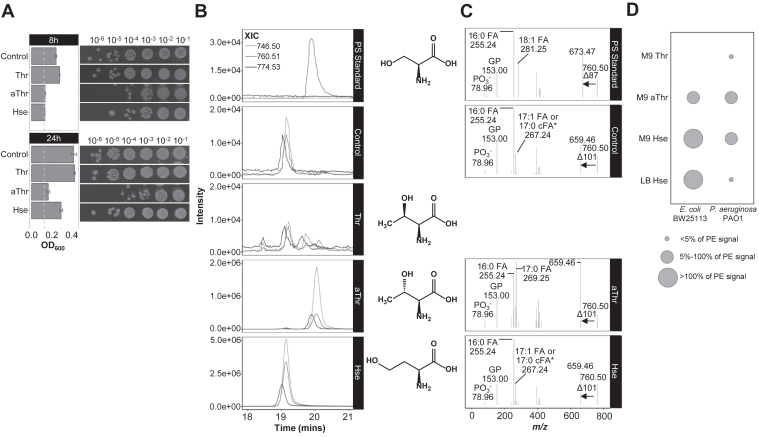
**Identification of phosphatidylhomoserine through metabolic substrate incorporation in *E. coli*.***A*, viability of *Escherichia coli* BW25113, grown in M9 minimal media supplemented with 0.1% (w/v) of substrates, _L_-threonine, _L_-*allo*-threonine, and _L_-homoserine, which are 14 Da larger than serine. Each spotting condition is represented with one technical spotting replicate within a set of biological replicates that includes two culture replicates, with three technical replicates for spotting. A total of three biological replicates were performed (*n* = 3). A_600_ bar graph was derived from the equivalent biological experiment and is representative of three biological replicates. *Left*: A_600_ (*Gray dashed line*: A_600_ of blank media control). *Right*: colony formation. *B*, extracted ion chromatograms (XIC) of three major ions of the unknown lipid class found endogenously in *E. coli*. Lipids analyzed were derived from *E. coli* BW25113 grown in M9 minimal media, supplemented with the substrates, _L_-threonine, _L_-*allo*-threonine, and _L_-homoserine (0.1% (w/v)). Data represented are from 8-h cultures. Treatment with _L_-*allo*-threonine and _L_-homoserine resulted in the accumulation of lipids at distinct retention times, with the latter co-eluting with the endogenous lipids found in *E. coli*. _L_-threonine treatment did not result in any incorporation, evident from the signals in the XIC. *C*, MS^2^ spectra of *E. coli* BW25113 lipids with *m/z* 760.50 under control (endogenous) and substrate-incorporated conditions. Both _L_-*allo*-threonine and _L_-homoserine incorporated lipids which formed a neutral loss of 101 Da. The XIC and spectra were derived from one set of experiment and are representative of three biological replicates. *D*, organism- and media-dependent incorporation of substrates. Both *E. coli* BW25113 and *P. aeruginosa* PAO1 were cultured in M9 minimal media and treated with the various substrates, _L_-threonine, _L_-*allo*-threonine, and _L_-homoserine (0.1% (w/v)). _L_-homoserine incorporation was additionally determined when bacteria were grown in rich media, LB. Incorporation of these substrates into phospholipids was determined and expressed relative to PE levels. aThr, _L_-*allo*-threonine; cFA, cyclopropane fatty acid; Da, Dalton; FA, fatty acid; GP, glycerophosphate; Hse, _L_-homoserine; LB, Luria-Bertani broth (Miller); *m/z*, mass-to-charge ratio; M9, M9 minimal media; MS^2^, tandem mass spectrometry; OD_600_, optical density at wavelength 600 nm; PE, phosphatidylethanolamine; PO_3_^-^, phosphate; PS standard, phosphatidylserine standard; Thr, _L_-threonine; XIC, extracted ion chromatogram. ∗The analytical method does not distinguish between mono-unsaturated fatty acid and cyclopropane fatty acid.

To determine if *E. coli* can incorporate threonine or other metabolites which share the same molecular weight directly into its phospholipids, we next established bacterial culture conditions using M9, a salt-based minimal media, to introduce specific substrates in a defined fashion (Figs. 2*A* and S1). This approach built on the ability of bacteria to transport amino acids and derivatives using common transporters (41). By providing the appropriate substrate in excess, we postulate the bacterium will accumulate the lipid of interest through its endogenous lipid biosynthetic machinery.

When *E. coli* was cultured in the defined minimal media containing glucose as the sole exogenous metabolite, the unknown feature was detected (Fig. 2*B*, control), suggesting the bacterium was able to produce this lipid from endogenous substrates. In contrast, the unknown feature was not detected in *P. aeruginosa* cultured in the same defined media (Fig. S2, control). Considering the substrates for the lipid formation were endogenously formed, we tested the bacterium’s ability to incorporate _L_-threonine (Thr), _L_-*allo*-threonine (aThr), and _L_-homoserine (Hse) into its lipids. Threonine is a proteinogenic amino acid, while _L_-*allo*-threonine is a rare isomer, which can be metabolized by *E. coli* (42). Homoserine, on the other hand, is a nonproteinogenic amino acid and a metabolic intermediate for the synthesis of methionine and threonine. Considering the multiple pathways an amino acid can be involved in, each metabolite was supplied in excess to ensure an adequate supply of substrates for lipid incorporation. At high concentration (15 mM (equivalent to 0.18% (w/v))), _L_-homoserine was previously shown to restrict the growth of *E. coli* in minimal media (43). In this study, *E. coli* cultured in minimal media with 0.1% (w/v) _L_-homoserine and _L_-*allo*-threonine exhibited reduced growth (Figs. 2*A* and S1). However, in both conditions, the bacterium was able to form colonies, and sufficient biomass was obtained for lipid analysis.

The lipid composition of *E. coli* treated with these substrates was analyzed using LC/MS^2^. Figure 2*B* showed the extracted ion chromatograms (XIC), based on the major ions of the endogenous lipid found in *E. coli*. Interestingly, *E. coli* treated with _L_-*allo*-threonine and _L_-homoserine, but not _L_-threonine, led to accumulation of lipids detected at distinct retention times. The lipids formed from _L_-*allo*-threonine treatment co-eluted with the PS standard, while the lipids formed from _L_-homoserine treatment co-eluted with the endogenous lipid detected in *E. coli*. In contrast, no observable accumulation of lipids was detected in the presence of _L_-threonine. MS^2^ profiles of the respective major ions formed from _L_-*allo*-threonine and _L_-homoserine incorporation revealed the signature of 101 Da neutral loss, as well as the expected sum fatty acid composition of the endogenous lipid (33 carbons, with one double bond or one cyclopropane fatty acid) (Fig. 2*C*). Based on the combination of retention time and spectra similarity, we concluded that the endogenous lipid produced by *E. coli* is PHS. The possibility of the *E. coli* lipid being PT was ruled out based on the distinct retention time of lipids accumulation in *P. aeruginosa* PAO1 treated with _L_-threonine (Fig. S2).

Indeed, *P. aeruginosa* PAO1 exhibited differences in incorporation of the different substrates, in contrast to *E. coli* (Fig. 2*D*). Unexpectedly, although PHS was not detected in *P. aeruginosa* PAO1 cultured in rich media (Fig. 1*C*) or minimal media (Fig. S2*B*, control), we observed an accumulation of PHS in *P. aeruginosa* PAO1 grown in minimal media supplemented with _L_-homoserine (Fig. 2*D*). This accumulation was not present when the bacterium was grown in rich media supplemented with _L_-homoserine, unlike in *E. coli*, which was able to accumulate PHS in both media types (Fig. 2*D*). Since _L_-homoserine is an intermediate in essential amino acid synthesis, its availability may be the limiting factor in PHS formation. To test the effects of _L_-homoserine concentration on incorporation into phospholipids, *E. coli* was cultured with varying concentrations of the substrate. The reduction of growth rate of the bacterium is dependent on the concentration of _L_-homoserine, consistent with earlier reports (43) (Fig. S3*A*). Nonetheless, the concentration of _L_-homoserine supplemented exogenously to *E. coli* influenced the degree of incorporation into phospholipids to form PHS (Fig. S3*B*). The bacterium, despite the slowed growth at higher _L_-homoserine concentration, was able to accumulate high levels of PHS, suggesting the formation of PHS is independent of the growth rate.

### Substrate promiscuity of *Ec*PssA is responsible for the formation of PHS

**Figure 3:**
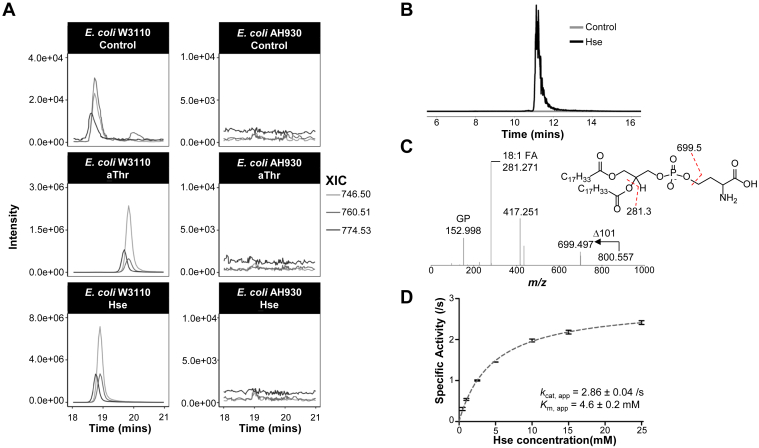
**Formation of phosphatidylhomoserine through the activity of *Ec*PssA.***A*, PS biosynthetic pathway is responsible for the incorporation of PHS in *Escherichia coli*. Extracted ion chromatograms of major PHS ions in WT *E. coli* W3110 (*top left panel*), which was used to further probe for their presence in both WT and AH930 Δ*pssA* mutant, untreated and treated with 0.1% (w/v) _L_-homoserine and _L_-*allo*-threonine. WT *E. coli* W3110 was able to produce PHS and PaT in the presence of _L_-homoserine and _L_-*allo*-threonine, while the signals were close to noise level for *E. coli* Δ*pssA* mutant (AH930), under all the conditions. *B*, extracted ion chromatogram of 18:1/18:1 PHS produced in a reaction mixture containing *Ec*PssA (*black line*) or lacking the enzyme (*gray line*). *C*, MS^2^ spectra of enzymatically synthesized 18:1/18:1 PHS and its chemical structure. *D*, Michaelis–Menten kinetics of *Ec*PssA with _L_-homoserine. Initial reaction velocities were measured using varying concentrations of _L_-homoserine (0.5, 1.0, 2.5, 5.0, 10.0, 15.0, and 25.0 mM) in the presence of 0.3 mM 18:1/18:1 CDP-DAG at 25 °C. Data points represent the mean ± SD (*n* = 3). /s, per second; aThr, _L_-*allo*-threonine; CDP-DAG, cytidine diphosphate diacylglycerol; *Ec*PssA, *E. coli* phosphatidylserine synthase; FA, fatty acid; GP, glycerophosphate; Hse, _L_-homoserine; *k*_cat_, app, apparent catalytic constant; *K*_m_, app: apparent Michaelis constant; *m/z*, mass-to-charge ratio; MS^2^, tandem mass spectrometry; PaT, phosphatidyl-*allo*-threonine; PHS, phosphatidylhomoserine; PS, phosphatidylserine; XIC, extracted ion chromatogram.

We next investigated the enzymatic machinery responsible for PHS formation. _L_-homoserine and _L_-threonine differ structurally in terms of the position of the hydroxy group. Since *E. coli* does not produce PT, and lacks a *Tg*PTS homolog, we ruled out the possibility of the activity of a PTS-like enzyme. Kanfer and Kennedy reported the limited effects on PssA activity when threonine was used as a substrate (11), but this does not exclude the possibility the involvement of PssA in PHS synthesis. Homoserine harbors a free terminal hydroxyl-group, which is present in serine. Indeed, this terminal hydroxy-group is involved in the condensation of serine with CDP-DAG to form PS. We hence questioned if *Ec*PssA is responsible for the synthesis of PHS. A mutant of PssA (*E. coli* strain AH930) was generated by DeChavigny *et al.*, which lacked PE and required divalent metal ions, including Mg^2+^, to grow (18). LC/MS analysis of the lipid extracts of *E. coli* strain AH930 confirmed the absence of PHS, in contrast to its parental WT strain, *E. coli* W3110 (Fig. 3*A*, top panels). To determine if the incorporation of the exogenously introduced substrates was dependent on *Ec*PssA, both WT *E. coli* W3110 and Δ*pssA* mutant, *E. coli* AH930, were treated with _L_-*allo*-threonine and _L_-homoserine. WT *E. coli* W3110 was able to incorporate both substrates (Fig. 3*A*, left panels) to form phosphatidyl-*allo*-threonine (PaT) and PHS, consistent with the findings using *E. coli* strain BW25113 (Fig. 2*B*). *E. coli* AH930, on the other hand, did not accumulate any PHS or PaT (Fig. 3*A*, right panels). These results confirmed PHS is formed endogenously in *E. coli* through the activity of *Ec*PssA, using _L_-homoserine as a substrate. Additionally, the substrate preference of *Ec*PssA also extends to _L_-*allo*-threonine.

To further confirm the utilization of _L_-homoserine by *Ec*PssA as a substrate, enzymatic assays using purified *Ec*PssA was performed. Incubation of _L_-homoserine and 18:1/18:1 CDP-DAG as substrates in the presence of *Ec*PssA led to the formation of 18:1/18:1 PHS, as confirmed by LC/MS^2^ (Fig. 3, *B* and *C*). Using a spectrophotometric-based kinetic assay, we found that *Ec*PssA displayed an apparent *k*_cat_ (*k*_cat, app_) of 2.86 ± 0.04 s^−1^ and apparent *K*_m_ (*K*_m, app_) of 4.6 ± 0.02 mM (Fig. 3*D*). In comparison, *k*_cat_ and *K*_m_ for _L_-serine is 69.7 ± 7.5 s^−1^ and 0.14 ± 0.03 mM respectively, as previously determined (44). These findings collectively confirmed *Ec*PssA can use _L_-homoserine as a substrate, albeit with low efficiency, and is responsible for the production of endogenous PHS.

### Decarboxylation of PHS to form PPA endogenously in Enterobacteriaceae

**Figure 4:**
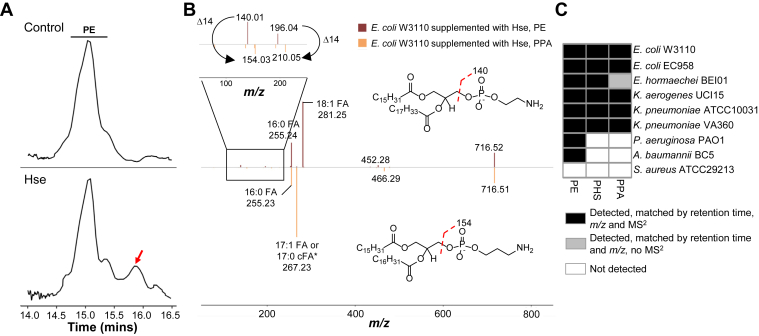
**Endogenous formation of phosphatidylpropanolamine.***A*, representative chromatogram of *Escherichia coli* W3110 cultured without and with 0.1% (w/v) _L_-homoserine, focusing on the elution region of phosphatidylethanolamine (PE). Bacteria cultured with _L_-homoserine accumulated an additional peak eluting after PE (*red arrow*). *B*, decarboxylation of PHS in *E. coli* treated with 0.1% (w/v) _L_-homoserine to form phosphatidylpropanolamine (PPA). MS^2^ spectra of PE (*top*, *dark red*) and PPA (*bottom, orange*). The decarboxylation of PHS was evident from the fragment ions with *m/z* 154.03 and 210.05 which are 14 Da larger than the phosphoethanolamine-linked fragment ions formed by PE. The data were derived from one set of experiment and are representative of three biological replicates. *C*, detection of endogenous PPA in Enterobacteriaceae at baseline. PPA was detected in Enterobacteriaceae and not in other bacterial species. cFA, cyclopropane fatty acid; Da, Dalton; FA, fatty acid; Hse, _L_-homoserine; *m/z*, mass-to-charge ratio; MS^2^, tandem mass spectrometry; PE, phosphatidylethanolamine; PHS, phosphatidylhomoserine; PPA, phosphatidylpropanolamine. ∗The analytical method does not distinguish between mono-unsaturated fatty acid and cyclopropane fatty acid.

The ability of *E. coli* to synthesize other phospholipids is not restricted to PHS. Interestingly, in WT *E. coli* treated with _L_-homoserine, we detected an accumulation of another peak that closely eluted with PE (Fig. 4*A*), which was confirmed using MS^2^ to be the decarboxylated form of PHS, PPA (Fig. 4*B*, bottom). PPA forms fragment ions (*m/z* 154.03 and 210.05), which are 14 Da larger than the dehydrated phosphoethanolamine and glycerophosphoethanolamine fragments (*m/z* 140.01 and *m/z* 196.04) (45) formed from PE (Fig. 4*B*, top). To determine if PPA is produced in bacteria endogenously, the lipidomics data of Enterobacteriaceae, *P. aeruginosa* and *A. baumannii* were screened for the headgroup diagnostic ions, parent ion mass, and retention time of PPA. PPA was detected in all Enterobacteriaceae analyzed at very low level, but not in *P. aeruginosa* and *A. baumannii* (Fig. 4*C*), which is consistent with the absence of PHS in the latter bacterial species. The results overall suggested *E. coli* and other Enterobacteriaceae can decarboxylate PHS, albeit at a lower efficiency in contrast to the formation of PE from PS.

### Effects of _L_-homoserine on global phospholipidome of *E. coli*

**Figure 5:**
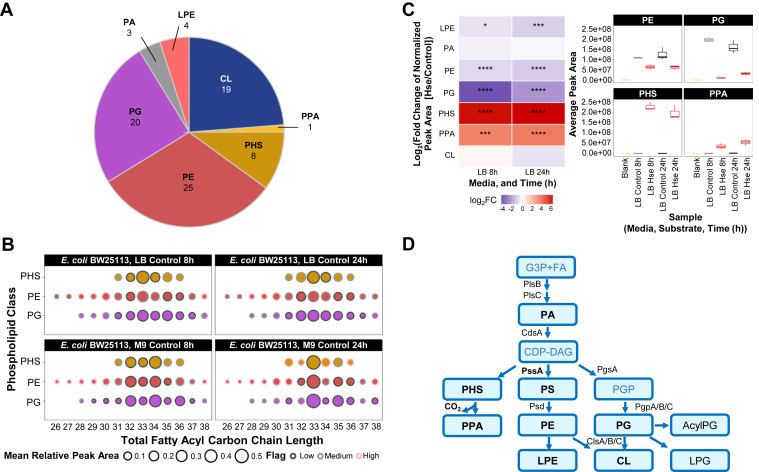
**Baseline phospholipidome of *Escherichia coli* and the effects of _L_-homoserine on phospholipid composition.***A*, number of phospholipids detected and characterized in *E. coli* BW25113 grown in LB- and M9-based media. The numerical values represent the number of ions detected and characterized, with MS^2^ data for each ion from at least one of the samples analyzed in *E. coli* BW25113 grown in LB- and M9-based media for 8 and 24 h. Each ion is annotated at the species level (sum of carbon atoms and double bonds). *B*, relative distribution of total carbon numbers of fatty acyl chains found in the diacylated phospholipids, PG, PE, and PHS. The size of the circles denoted the relative peak area of a specific carbon number (sum of both fatty acyl chains), normalized to all ions detected for a given phospholipid class. Colors of the borders of circles denote the coefficient of variance (CV, %) from three biological replicates. *C*, changes in phospholipid class distribution during culture in LB supplemented with 0.1% (w/v) _L_-homoserine. Heatmap represented the fold change of the proportion of the peak areas attributed by each phospholipid class, relative to the total peak areas of all phospholipid classes detected. Data were generated from three biological replicates, and statistical analysis was performed using Student’s *t* test, with Bonferroni correction. Symbols represented adjusted *p*-values: ∗*p* < 0.05, ∗∗*p* < 0.01, ∗∗∗*p* < 0.001, ∗∗∗∗*p* < 0.0001. Box plots representing the raw peak areas were presented for the four classes of lipids, PE, PG, PHS, and PPA, with the most significant differences when *E. coli* was treated with _L_-homoserine, to confirm these specific changes observed were not due to the normalization method. *D*, phospholipid pathway of *E. coli* updated based on this study. Phospholipid names in *black* were detected in this study, with bold representing phospholipid classes confirmed with MS^2^ characterization. Acyl phosphatidylglycerol (AcylPG) and lysophosphatidylglycerol (LPG) were detected but no MS^2^ data was available due to the low abundance. AcylPG, acyl phosphatidylglycerol; CDP-DAG, cytidine diphosphate diacylglycerol; CdsA, phosphatidate cytidylyltransferase; CL, cardiolipin; ClsA/B/C, cardiolipin synthase A/B/C; CO_2_, carbon dioxide; FA, fatty acyl; G3P, glycerol-3-phosphate; Hse, Homoserine; LB, Luria-Bertani broth (Miller); LPE, lysophosphatidylethanolamine; LPG, lysophosphatidylphosphatidylglycerol; M9, M9 minimal media; MS^2^, tandem mass spectrometry; PA, phosphatidic acid; PE, phosphatidylethanolamine; PG, phosphatidylglycerol; PGP, phosphatidylglycerol phosphate; PgpA/B/C, phosphatidylglycerophosphatase A/B/C; PgsA, phosphatidylglycerol phosphate (PGP) synthase; PHS, phosphatidylhomoserine; PlsB, glycerol-3-phosphate acyltransferase; PlsC, 1-acyl-sn-glycerol-3-phosphate acyltransferase; PPA, phosphatidylpropanolamine; PS, phosphatidylserine; Psd, phosphatidylserine decarboxylase; PssA, phosphatidylserine synthase.

Based on the combination of genetics, substrate incorporation, and LC/MS^2^ characterization, we identified PHS as a phospholipid class found in *E. coli* under baseline condition, when grown in both rich (LB) and minimal media. This also led us to uncover the presence of an even lower abundant lipid PPA, which is formed from the decarboxylation of PHS endogenously. Figure 5*A* summarized the classes of phospholipids and the associated number of molecular ions characterized in *E. coli* BW25113 when cultured in LB and M9 minimal media, using LC/MS^2^. The number of PHS and PPA ions detected is expectedly less than the major diacylated PE and PG (Fig. 5*A* and Table S1*A*), given their natural low abundance. The relative distribution of phospholipids with distinct fatty acyl chain length was dependent on the time point and the type of media (Fig. 5*B*). The major PHS in LB (both time points) as well as M9 minimal media at 24-h harbored a total of 33 carbons in the fatty acyl chains, and for bacteria grown in M9 minimal media at 8-h, the major forms comprised 32 and 34 carbons (Fig. 5*B* and Table S1*B*). Bacteria grown in LB at 8-h are in the deceleration phase of growth (Fig. S4) and it is expected there was accumulation of phospholipids with odd-numbered sum composition, since it is well-characterized that stationary phase bacterium accumulated fatty acids with 17-carbons and cyclopropane ring (35, 46, 47). In general, the carbon chain length of the highest abundance molecular ion of PHS mirrored that of both PE and PG (Fig. 5*B*).

To understand how the incorporation of _L_-homoserine affects global phospholipid remodeling in *E. coli*, we analyzed the lipids in *E. coli* BW25113, treated with _L_-homoserine. Cells were grown in LB, which was more favorable for growth of the bacterium under control and _L_-homoserine–treated conditions, in contrast to M9 minimal media (43) (Fig. S4). _L_-homoserine treatment led to pronounced accumulation of PHS and PPA at both 8-h and 24-h time points (Fig. 5*C* and Table S2). A concomitant and significant reduction in PE and PG levels was observed, suggesting potential substrate funneling of CDP-DAG, a common intermediate metabolite for the synthesis of PE, PG, and PHS. In contrast to the formation of PE from PS, which leads to undetectable levels of PS and high levels of PE, the relative level of PHS is higher than PPA, suggesting less efficient decarboxylation of PHS compared to PS. The phospholipid metabolic pathway of *E. coli*, based on the collective biochemical and genetics findings from this study, is summarized in Figure 5*D*.

## Discussion

In this work, we reported the discovery and characterization of PHS and PPA, two new phospholipid members in the baseline lipidome of *E. coli*, which were also detected in other Enterobacteriaceae species analyzed. We further provided genetic and biochemical evidence that PHS is formed through the activity of *Ec*PssA. Treatment of *E. coli* with exogenous _L_-homoserine led to the accumulation of PHS and its decarboxylated form, PPA, as well as a concomitant reduction of PG and PE. To date, PHS has only been reported in *Mycoplasma fermentans*, with its structure resolved based on the analysis of hydrolyzed products (48). Our works provided the first structural characterization of intact PHS molecules and the biochemical pathway involved in its endogenous production in *E. coli*. Endogenous production of PPA, on the other hand, has yet to be reported in any organism.

In *E. coli*, the main enzymatic activity of PssA is the production of PS using CDP-DAG and serine as the primary substrates (11). Using an *in vitro* purified enzyme system, *Ec*PssA was able to incorporate phosphoglycerol to form PG, albeit at low levels, but it lacked activity towards threonine (11). We provided biochemical evidence that *Ec*PssA is able to incorporate _L_-homoserine into PHS. In addition, at the cellular level, the bacterium is able to use both _L_-homoserine and _L_-*allo*-threonine, to form PHS and PaT, respectively. Both _L_-homoserine and _L_-*allo*-threonine are isomers of _L_-threonine, demonstrating that *Ec*PssA has restricted activity towards _L_-threonine possibly due to its stereochemistry (44). Homoserine differs from serine by an insertion of an additional CH_2_ unit, but it retains a free terminal hydroxy group, the functional group in serine involved in condensation with CDP-DAG. The catalytic efficiency (*k*_cat_/*K*_m_) of *Ec*PssA for _L_-serine and _L_-homoserine differ significantly, with _L_-serine being the preferred substrate by approximately 270-fold. This difference likely stems from the confined nature of the amino acid–binding pocket, as suggested by the serine-bound model of *Ec*PssA, which reveals multiple ionic and polar interactions that are critical for recognition of the cognate substrate and precise positioning of its nucleophilic hydroxyl group (44). The intracellular concentration of serine in WT *E. coli* has been reported to be ∼70 μM (49), whereas the concentration of homoserine has, to our knowledge, not been quantified, likely due to its low abundance. Taken together, the kinetic parameters of *Ec*PssA support a physiological preference for _L_-serine over _L_-homoserine. Interestingly, *E. coli* was able to incorporate _L_-*allo*-threonine but not _L_-threonine, which are diastereomers. _L_-*allo*-threonine and _L_-threonine differ in the spatial position of the methyl group bound to the β-carbon, which may be a possible steric hindrance for the interaction of _L_-threonine with the active site of *Ec*PssA. This postulation will require detailed structural analysis of purified *Ec*PssA and its interactions with potential substrates. PHS had been produced *in vitro* with a reconstituted enzymatic reaction using cabbage or *Streptomyces* sp. PLD, with phosphatidylcholine and homoserine as substrates (50). Although *E. coli* harbors PLD activities (30, 51, 52), our works ruled out the possibility of PHS formation through other PLD, based on our findings that Δ*pssA* mutant does not incorporate _L_-homoserine into its lipids.

The specificity of incorporation is dependent on the organism, as well as the growth media. This is evident from our findings that unlike *E. coli*, *P. aeruginosa* does not produce detectable levels of PHS at baseline in both LB and M9 minimal media. This is not due to the sequence differences in the PS synthase in the two bacteria species, since *P. aeruginosa* was able to incorporate _L_-homoserine into its phospholipids when grown in M9 minimal media supplemented with the substrate. The formation of PHS in *E. coli* is dependent on the concentration of _L_-homoserine, suggesting that PHS synthesis is regulated by substrate availability. Homoserine is an endogenous metabolic intermediate which is formed from aspartate and serves as a precursor for the amino acids, threonine and methionine. Therefore, it is not surprising to observe low levels of PHS in *E. coli* and its absence in *P. aeruginosa*, if _L_-homoserine turnover and hence availability differs between bacterial species. Moreover, cultivation in rich and minimal media creates distinct pools of metabolites, which influence both the metabolic state of the bacterium and substrate availability (53, 54, 55). Interestingly, _L_-homoserine at high concentration (15 mM (equivalent to 0.18% (w/v))) leads to growth restriction of *E. coli* when grown in minimal media, but not rich media (43). Despite the growth restrictions in minimal media, *E. coli* was able to produce high levels of PHS in the presence of exogenous _L_-homoserine, suggesting *Ec*PssA remained active despite the impeded growth rate. Whether the incorporation of _L_-homoserine to phospholipids is linked to restricted growth or served as a protective mechanism to buffer this metabolite will warrant in-depth investigation.

The substrate promiscuity of phospholipid metabolizing enzymes is clearly not restricted to PssA, evident from the production of PPA when *E. coli* was treated with _L_-homoserine. PPA is likely to be formed through decarboxylation of PHS, through the activity of Psd. In contrast, PPA had been reported in eukaryotic system through the incorporation of propanolamine as a substrate in cell cultures (56, 57, 58). In the budding yeast, *Saccharomyces cerevisiae*, PPA, formed from the exogenous provision of propanolamine to the cells can rescue the growth of *psd1/psd2* double mutant which lacked PE (58). PHS, produced *in vitro* using PLD, had been shown to weakly activate the PS flippase, Atp8a1 (59). The precise functions of endogenously produced PHS and PPA in *E. coli*, however, will require further investigation. Nonetheless, the renewed knowledge on the substrate promiscuity of enzymes involved in phospholipid synthesis, along with the structural specificity of the enzyme-substrate relationship could potentially guide the design of novel probes for metabolic labeling (60, 61) to facilitate future studies on lipid metabolism, transport, distribution, and functions.

Cellular lipidome is evidently shaped by both endogenous and exogenous chemicals, which can be directly incorporated into lipids (30, 31). This is demonstrated in the present study, where _L_-homoserine and _L_-*allo*-threonine were incorporated into phospholipids in *E. coli*. Nonclassical lipids can be produced through various mechanisms: (i) broad substrate specificity of known enzymes, (ii) presence of domains with additional activities in known enzymes, (iii) presence of unknown enzymes, and (iv) nonenzymatic reactions. As a unicellular organism which resides in both the natural physical environment and human hosts (62), *E. coli* is exposed to varying environments with different biochemicals. However, laboratory media for cultivating bacteria such as LB (63) had been tailored towards promoting growth but does not truly reflect the environment the organism is exposed to. Our works provided the first cellular evidence that *E. coli* is able to produce other lipids endogenously using its existing known biochemical resources, specifically, PssA and endogenous homoserine. A more exacting picture of the global lipidome of *E. coli* and other bacteria species will require more systematic analysis involving conditions that mimic the external growth environment of the bacteria, including pH, temperature, and other exogenous compounds. With a more thorough understanding of the biochemistry of *E. coli* and other microbes, they can further serve as powerful biological factories for the production of atypical lipids. In this study, the metabolic plasticity of *E. coli* had served as an important source of lipid production which facilitated the characterization of PHS and PPA.

This work combined analytical chemistry, genetics, and biochemistry to confirm the structure and biosynthetic mechanism of PHS, a previously uncharacterized phospholipid class in *E. coli*. However, two bioanalytical limitations exist. Firstly, there is a lack of pertinent standards for the nonclassical lipids, as well as phospholipids with cyclopropane fatty acyls, which is common in *E. coli* and other bacteria (35, 46, 64, 65). It is hence important to note that the presented values based on peak areas do not reflect the actual lipid concentration, since peak areas depend on both concentration and ionization efficiency of the lipid. Furthermore, bacterial cell shape and biomass change during growth (66, 67). Hence, the pairwise (control vs treatment) comparison of the relative levels of lipid classes were based on normalization to the total phospholipids detected to control the amounts of materials. Raw peak areas were presented to ensure the observed changes in PHS, PPA, PG, and PE were not due to the normalization method or lack of internal standards. Secondly, MS^2^ was performed using collision-induced dissociation, which lacked the resolving power for double bonds position, and fatty acyl chain modifications including branch chain and cyclopropane rings. Hence, to avoid misreporting of lipid structures, we had examined the lipid composition at the carbon chain length level within each given class, and due consideration was given for annotating fatty acids sharing the same *m/z* for monounsaturated and cyclopropane forms. The fine structures of the fatty acyl constituents can be further resolved using alternative methods, including ultraviolet photodissociation mass spectrometry (35, 64), ozone dissociation (68) or Paternò-Büchi derivatization, and LC/MS^2^ (69). Certainly, more work will be required to establish both qualitative and quantitative bacterial lipidomics and their applications in research to deepen our current understanding of bacterial lipid metabolism and functions.

## Experimental procedures

### Bacteria strains

Bacteria and strains used in this study include *E. coli* BW25113 (KEIO library, WT), *P*. *aeruginosa* PAO1 and *S*. *aureus* ATCC 29213 (from Kevin Pethe), *E. coli* W3110 and AH930 (Δ*pssA* mutant) (from William Dowhan, (18)), *E. coli* EC958 (70), *K*. *pneumoniae* ATCC10031 (ATCC), *K. pneumoniae* VA360, *A*. *baumannii* BC5, *Escherichia hormaechei* BEI01, *K*. *aerogenes* UCI15 (BEI resources).

### General bacteria culture conditions

Bacteria precultures were grown overnight in LB broth (Miller), incubated at 37 °C with shaking at 150 rpm in Infors HT Ecotron incubator. The optical density (OD) at wavelength 600 nm (OD_600_) of the overnight cultures was measured using Thermo Scientific Multiskan GO Microplate Spectrophotometer. The cultures were then inoculated in LB at an OD_600_/ml of 0.01 or 0.05 for growth at 37 °C with shaking at 150 rpm, up to the respective time points, for sampling. For W3110 and AH930, the media were supplemented with 23.4 mM MgCl_2_.

For initial characterization of lipidome of *E. coli*, *K. pneumoniae*, *K. aerogenes*, *A. baumannii*, *P. aeruginosa*, and *S. aureus*, cultures were sampled at 24-h. The cultures were centrifuged at 3000*g* for 10 min at 4 °C to remove the media. The samples were washed twice in cold M9 wash media (1× commercial M9 without any supplementation of salt or carbon source), and the final pellets were snap frozen with liquid nitrogen and stored at −80 °C.

### Substrate incorporation for WT *E. coli* BW25113 and *P. aeruginosa* PAO1

Overnight bacterial precultures were centrifuged at 3000*g* for 10 min at room temperature to remove the rich media and washed once with M9 wash media. The pellets were resuspended in M9 minimal media containing 0.1 mM CaCl_2_, 2 mM MgSO_4_, 0.2% (w/v) glucose, and supplemented with the respective substrate (0.1%, w/v in water) to achieve a final A_600_/ml of 0.01. A starting concentration of 0.1% (w/v) of the substrates was used to ensure adequate supply for downstream biosynthesis pathways and potential incorporation into lipids. Substrates used include _L_-homoserine, _L_-threonine, and _L_-*allo*-threonine. The cultures were incubated at 37 °C with shaking at 150 rpm. After 8 h and 24 h, the OD_600_ of the cultures were measured, and sampling was performed for viability testing by spotting on LB agar. The choice of these time points was based on the logarithmic phase of *E. coli* BW25113 and *P. aeruginosa* PAO1 in minimal media supplemented with glucose (control) (8-h), and 24-h represented the stationary phase of growth. To determine the incorporation of _L_-homoserine in rich media, parallel cultures were set up and grown in LB or M9, supplemented with 0.1% (w/v) _L_-homoserine. Due to observable difference in growth rates, which is dependent on the bacterial species, culture media, and treatment, all sampling were fixed at these two time points.

For lipid analysis, the cultures were centrifuged at 3000*g* for 10 min at 4 °C to remove the media. The samples were washed once in cold M9 wash media, and the final pellets were snap frozen with liquid nitrogen and stored at −80 °C. Three sets of biological replicates were collected.

### Characterization of phospholipids and substrate incorporation in PssA *E. coli* mutant

Δ*pssA* mutant (AH930) and the parental WT (W3110) were grown in LB media supplemented with 23.4 mM MgCl_2_ using the above culture conditions. For substrate incorporation, the bacterial cultures were grown in LB media with 23.4 mM MgCl_2_ supplemented with the respective substrates at a final concentration of 0.1% (w/v in water) for 8 h and 24 h.

Sampling was performed by centrifugation at 3000*g* for 10 min at 4 °C, followed by two washes with M9 wash media. The final pellets were snap frozen with liquid nitrogen and stored at −80 °C. Three sets of biological replicates were collected, except for the substrate incorporation for 24 h which had two sets of biological replicates.

### Lipid extraction and analysis using LC/MS^2^

Lipids were extracted using a modified Bligh-Dyer method (71). Briefly, chloroform:methanol, 1:2 (v/v) were added to the bacterial pellets and the samples were incubated at 6 °C for 4 h. After the incubation, water and chloroform were added, followed by centrifugation for phase separation. The lower organic phase was collected and the upper phase was re-extracted with chloroform. The organic extracts were pooled and dried using either a SpeedVac (Labconco) or nitrogen stream. Lipid extracts were stored at −80 °C until further analysis.

For lipid characterization, mass spectrometry (MS) and MS^2^ were performed using a SCIEX TripleTOF 6600 quadrupole time-of-flight mass spectrometer, coupled to an Agilent 1290 liquid chromatography system. Normal phase chromatography (Intersil SIL column, 3 μm, 2.1 × 150 mm, GL Sciences) was used for separation, and lipids were analyzed in the negative electrospray ionization mode. The mobile phase A consisted of chloroform/methanol/ammonia hydroxide (89.5/10/0.5, v/v/v) and mobile phase B consisted of chloroform/methanol/water/ammonia (55/39/5.5/0.5). Automated MS^2^ analyses were performed using collision-induced dissociation in the information-dependent acquisition mode. The lipidomics minimal reporting checklist (72) is made available in Supplemental Material.

### LC/MS data analysis

For qualitative analysis, PeakView (SCIEX) was used for the visual review of spectra and extraction of ion chromatograms and MS^2^ spectra. Data lists were then exported into .txt files, and the XIC and MS^2^ spectra were plotted using the R package ggplot. All MS^2^ spectra and XICs presented are from one experiment and are representative of the three independent biological replicates. To determine the relative incorporation of substrates into phospholipids, peak areas of XIC of three most abundant molecular species of the respective lipid classes (if present) were integrated in PeakView and calculated as a percentage of the equivalent PE molecular species. For assessing the global lipidome, full dataset was extracted using mass spectrometry-data independent analysis software, version 4.8 (73). The relative peak areas of each sample were calculated by dividing the peak area of individual phospholipids by the sum of all identified phospholipids, after filtering lipids with less than 40% CV between the three biological batches. Fold change between conditions was computed, and statistical analysis was performed using Student’s *t* test with Bonferroni correction. For the relative distribution of phospholipids by their total fatty acyls carbon chain length within each phospholipid class, ions with the same carbon number within a phospholipid class were summed up and further normalized to the sum of all molecular ions of the given class. All statistical analysis and graphical production were performed using the R packages rstatix and ggplot (74, 75).

For lipid annotation, the following criteria must be fulfilled, with the exception for the currently characterized lipids, which were discussed in the results section: (i) retention time of lipid class, based on synthetic lipid standards (Avanti Polar Lipids), (ii) MS^2^ spectra matched for at least one sample of a given *E. coli* strain.

### Functional activity of purified *Ec*PssA using _L_-homoserine as a substrate

The activity of purified *E. coli* (K-12 strain MG1655) PssA (*Ec*PssA) using _L_-homoserine as a substrate was assessed using two approaches, as described by Lee *et al.* (44). Firstly, formation of PHS was determined using LC/MS using a reconstituted enzymatic assay, carried out following a modified approach described by Dowhan (76). The assay mixture was prepared by mixing 0.4 mM 18:1/18:1 CDP-DAG, 1 mM _L_-homoserine, and 1 mg/ml of bovine serum albumin. The reaction was initiated by adding 100 nM purified *Ec*PssA in buffer A, composed of 30 mM Tris–HCl, pH 7.5, 400 mM NaCl, 10% (v/v) glycerol, and 0.05% (w/v) *n*-octyl-β-_D_-glucoside (OG) and subsequently incubated at 37 °C for 30 min. The reaction was terminated using five volumes of chloroform/methanol (2:1, v/v) mixture, followed by vigorous vortexing. Lipid extraction was carried out using the Folch method (77) and analyzed using LC/MS analysis (44).

*Ec*PssA activity was further assessed by a coupled spectrophotometric assay. The assay quantitatively monitors the release of CMP from CDP-DAG and _L_-homoserine as substrates, based on the decrease in NADH absorbance at 340 nm, as previously described by Carman and Dowhan (78, 79). The reaction mixture contained 50 mM Tris–HCl, pH 7.5, 0.1 M KCl, 10 mM MgCl_2_, 1 mg/ml bovine serum albumin, 1 mM ATP, 1 mM phosphoenolpyruvate, 0.2 mM NADH, 0.2 U/ml of cytidine monophosphate kinase, 0.4 U/ml of pyruvate kinase, 0.4 U/ml of lactate dehydrogenase, 2.4 mM Triton X-100, and 0.3 mM 18:1/18:1 CDP-DAG substrate and varying concentration of _L_-homoserine in a total volume of 0.18 ml. Enzyme reactions were conducted at 25 °C and initiated by the addition of *Ec*PssA. The sample was briefly sonicated before absorbance measurements. The curve was fitted to the Michaelis–Menten equation using nonlinear regression in OriginPro 2021. All experiments were performed in triplicate, and data are presented as mean ± SD.

## Data availability

All data used in the article are available from the corresponding author upon reasonable request.

## Supporting information

This article contains supporting information.

## Conflict of interest

The authors declare that they have no conflicts of interests with the contents of this article.

## References

[1] Nikaido H. (2003). Molecular basis of bacterial outer membrane permeability revisited. Microbiol. Mol. Biol. Rev..

[2] Carty C.E., Ingram L.O. (1981). Lipid synthesis during the *Escherichia coli* cell cycle. J. Bacteriol..

[3] Raetz C.R., Dowhan W. (1990). Biosynthesis and function of phospholipids in *Escherichia coli*. J. Biol. Chem..

[4] Shibuya I. (1992). Metabolic regulations and biological functions of phospholipids in *Escherichia coli*. Prog. Lipid Res..

[5] Parsons J.B., Rock C.O. (2013). Bacterial lipids: metabolism and membrane homeostasis. Prog. Lipid Res..

[6] Zhang Y.M., Rock C.O. (2008). Membrane lipid homeostasis in bacteria. Nat. Rev. Microbiol..

[7] Dowhan W. (2013). A retrospective: use of *Escherichia coli* as a vehicle to study phospholipid synthesis and function. Biochim. Biophys. Acta.

[8] Raetz C.R. (1978). Enzymology, genetics, and regulation of membrane phospholipid synthesis in *Escherichia coli*. Microbiol. Rev..

[9] Sinensky M. (1974). Homeoviscous adaptation—a homeostatic process that regulates the viscosity of membrane lipids in *Escherichia coli*. Proc. Natl. Acad. Sci. U. S. A..

[10] Cronan J.E., Vagelos P.R. (1972). Metabolism and function of the membrane phospholipids of *Escherichia coli*. Biochim. Biophys. Acta.

[11] Kanfer J., Kennedy E.P. (1964). Metabolism and function of bacterial lipids: II. Biosynthesis of phospholipids in *Escherichia coli*. J. Biol. Chem..

[12] Lightner V.A., Larson T.J., Tailleur P., Kantor G.D., Raetz C.R., Bell R.M. (1980). Membrane phospholipid synthesis in *Escherichia coli*. Cloning of a structural gene (*plsB*) of the *sn*-glycerol-3-phosphate acyl/transferase. J. Biol. Chem..

[13] Coleman J. (1990). Characterization of *Escherichia coli* cells deficient in 1-acyl-*sn*-glycerol-3- phosphate acyltransferase activity. J. Biol. Chem..

[14] Coleman J. (1992). Characterization of the *Escherichia coli* gene for 1-acyl-*sn*-glycerol-3-phosphate acyltransferase (*plsC*). Mol. Gen. Genet..

[15] Ganong B.R., Leonard J.M., Raetz C.R. (1980). Phosphatidic acid accumulation in the membranes of *Escherichia coli* mutants defective in CDP-diglyceride synthetase. J. Biol. Chem..

[16] Ganong B.R., Raetz C.R. (1982). Massive accumulation of phosphatidic acid in conditionally lethal CDP-diglyceride synthetase mutants and cytidine auxotrophs of *Escherichia coli*. J. Biol. Chem..

[17] Kiyasu J.Y., Pieringer R.A., Paulus H., Kennedy E.P. (1963). The biosynthesis of phosphatidylglycerol. J. Biol. Chem..

[18] DeChavigny A., Heacock P.N., Dowhan W. (1991). Sequence and inactivation of the *pss* gene of *Escherichia coli*. Phosphatidylethanolamine may not be essential for cell viability. J. Biol. Chem..

[19] Raetz C.R. (1976). Phosphatidylserine synthetase mutants of *Escherichia coli*. Genetic mapping and membrane phospholipid composition. J. Biol. Chem..

[20] Hawrot E., Kennedy E.P. (1975). Biogenesis of membrane lipids: mutants of *Escherichia coli* with temperature-sensitive phosphatidylserine decarboxylase. Proc. Natl. Acad. Sci. U. S. A..

[21] Hawrot E., Kennedy E.P. (1976). Conditional lethal phosphatidylserine decarboxylase mutants of *Escherichia coli*. Mapping of the structural gene for phosphatidylserine decarboxylase. Mol. Gen. Genet..

[22] Hawrot E., Kennedy E.P. (1978). Phospholipid composition and membrane function in phosphatidylserine decarboxylase mutants of *Escherichia coli*. J. Biol. Chem..

[23] Lu Y.H., Guan Z., Zhao J., Raetz C.R. (2011). Three phosphatidylglycerol-phosphate phosphatases in the inner membrane of *Escherichia coli*. J. Biol. Chem..

[24] Tan B.K., Bogdanov M., Zhao J., Dowhan W., Raetz C.R., Guan Z. (2012). Discovery of a cardiolipin synthase utilizing phosphatidylethanolamine and phosphatidylglycerol as substrates. Proc. Natl. Acad. Sci. U. S. A..

[25] Guo D., Tropp B.E. (2000). A second *Escherichia coli* protein with CL synthase activity. Biochim. Biophys. Acta.

[26] Tunaitis E., Cronan J.E. (1973). Characterization of the cardiolipin synthetase activity of *Escherichia coli* envelopes. Arch. Biochem. Biophys..

[27] Khersonsky O., Tawfik D.S. (2010). Enzyme promiscuity: a mechanistic and evolutionary perspective. Annu. Rev. Biochem..

[28] Larson T.J., Dowhan W. (1976). Ribosomal-associated phosphatidylserine synthetase from *Escherichia coli*: purification by substrate-specific elution from phosphocellulose using cytidine 5'-diphospho-1,2-diacyl-*sn*-glycerol. Biochemistry.

[29] Jeucken A., Molenaar M.R., van de Lest C.H.A., Jansen J.W.A., Helms J.B., Brouwers J.F. (2019). A comprehensive functional characterization of *Escherichia coli* lipid genes. Cell Rep..

[30] Jeucken A., Helms J.B., Brouwers J.F. (2018). Cardiolipin synthases of *Escherichia coli* have phospholipid class specific phospholipase D activity dependent on endogenous and foreign phospholipids. Biochim. Biophys. Acta Mol. Cell Biol. Lipids.

[31] Shibuya I., Yamagoe S., Miyazaki C., Matsuzaki H., Ohta A. (1985). Biosynthesis of novel acidic phospholipid analogs in *Escherichia coli*. J. Bacteriol..

[32] Wang X., Xia K., Yang X., Tang C. (2019). Growth strategy of microbes on mixed carbon sources. Nat. Commun..

[33] Brügger B. (2014). Lipidomics: analysis of the lipid composition of cells and subcellular organelles by electrospray ionization mass spectrometry. Annu. Rev. Biochem..

[34] Han X., Gross R.W. (2022). The foundations and development of lipidomics. J. Lipid Res..

[35] Kralj T., Nuske M., Hofferek V., Sani M.-A., Lee T.-H., Separovic F. (2022). Multi-omic analysis to characterize metabolic adaptation of the *E. coli* lipidome in response to environmental stress. Metabolites.

[36] Hines K.M., Xu L. (2019). Lipidomic consequences of phospholipid synthesis defects in *Escherichia coli* revealed by HILIC-ion mobility-mass spectrometry. Chem. Phys. Lipids.

[37] Garrett T.A., Raetz C.R., Richardson T., Kordestani R., Son J.D., Rose R.L. (2009). Identification of phosphatidylserylglutamate: a novel minor lipid in *Escherichia coli*. J. Lipid Res..

[38] Garrett T.A., Raetz C.R.H., Son J.D., Richardson T.D., Bartling C., Guan Z. (2011). Non-enzymatically derived minor lipids found in *Escherichia coli* lipid extracts. Biochim. Biophys. Acta Mol. Cell Biol. Lipids.

[39] Arroyo-Olarte R.D., Brouwers J.F., Kuchipudi A., Helms J.B., Biswas A., Dunay I.R. (2015). Phosphatidylthreonine and lipid-mediated control of parasite virulence. PLoS Biol..

[40] Hajeyah A.A., Protty M.B., Paul D., Costa D., Omidvar N., Morgan B. (2024). Phosphatidylthreonine is a procoagulant lipid detected in human blood and elevated in coronary artery disease. J. Lipid Res..

[41] Templeton B.A., Savageau M.A. (1974). Transport of biosynthetic intermediates: homoserine and threonine uptake in *Escherichia coli*. J. Bacteriol..

[42] Liu J.-Q., Dairi T., Itoh N., Kataoka M., Shimizu S., Yamada H. (1998). Gene cloning, biochemical characterization and physiological role of a thermostable low-specificity _L_-threonine aldolase from *Escherichia coli*. Eur. J. Biochem..

[43] Kotre A.M., Sullivan Stephen J., Savageau Michael A. (1973). Metabolic regulation by homoserine in *Escherichia coli* B/r. J. Bacteriol..

[44] Lee E., Cho G., Kim J. (2024). Structural basis for membrane association and catalysis by phosphatidylserine synthase in *Escherichia coli*. Sci. Adv..

[45] Han X., Gross R.W. (1995). Structural determination of picomole amounts of phospholipids via electrospray ionization tandem mass spectrometry. J. Am. Soc. Mass Spectrom..

[46] Berezhnoy N.V., Cazenave-Gassiot A., Gao L., Foo J.C., Ji S., Regina V.R. (2022). Transient complexity of *E. coli* lipidome is explained by fatty acyl synthesis and cyclopropanation. Metabolites.

[47] Wang A.-Y., Cronan J.E. (1994). The growth phase-dependent synthesis of cyclopropane fatty acids in *Escherichia coli* is the result of an RpoS(KatF)-dependent promoter plus enzyme instability. Mol. Microbiol..

[48] Deutsch J., Salman M., Rottem S. (1995). An unusual polar lipid from the cell membrane of *Mycoplasma fermentans*. Eur. J. Biochem..

[49] Bennett B.D., Kimball E.H., Gao M., Osterhout R., Van Dien S.J., Rabinowitz J.D. (2009). Absolute metabolite concentrations and implied enzyme active site occupancy in Escherichia coli. Nat Chem Biol.

[50] Shuto S., Imamura S., Fukukawa K., Sakakibara H., Murase J. (1987). A facile one-step synthesis of phosphatidylhomoserines by phospholipase D-catalyzed transphosphatidylation. Chem. Pharm. Bull. (Tokyo).

[51] Cole R., Benns G., Proulx P. (1974). Cardiolipin specific phospholipase D activity in *Escherichia coli* extracts. Biochim. Biophys. Acta.

[52] Cole R., Proulx P. (1975). Phospholipase D activity of Gram-negative bacteria. J. Bacteriol..

[53] Tramontano M., Andrejev S., Pruteanu M., Klunemann M., Kuhn M., Galardini M. (2018). Nutritional preferences of human gut bacteria reveal their metabolic idiosyncrasies. Nat. Microbiol..

[54] Liu Y.K., Kuo H.C., Lai C.H., Chou C.C. (2020). Single amino acid utilization for bacterial categorization. Sci. Rep..

[55] Moreno R., Rojo F. (2023). The importance of understanding the regulation of bacterial metabolism. Environ. Microbiol..

[56] Kovacs P., Kohidai L., Csaba G. (1997). Effect of 3-amino-1-propanol on the phosphatidylinositol (PI) and glycosyl phosphatidylinositol (GPI) systems of *Tetrahymena*. Comparative Biochemistry and Physiology Part C: pharmacology. Toxicol. Endocrinol..

[57] Glaser M., Ferguson K.A., Vagelos P.R. (1974). Manipulation of the phospholipid composition of tissue culture cells. Proc. Natl. Acad. Sci. U. S. A..

[58] Storey M.K., Clay K.L., Kutateladze T., Murphy R.C., Overduin M., Voelker D.R. (2001). Phosphatidylethanolamine has an essential role in *Saccharomyces cerevisiae* that is independent of its ability to form hexagonal phase structures. J. Biol. Chem..

[59] Paterson J.K., Renkema K., Burden L., Halleck M.S., Schlegel R.A., Williamson P. (2006). Lipid specific activation of the murine P_4_-ATPase Atp8a1 (ATPase II). Biochemistry.

[60] Ignacio B.J., Bakkum T., Bonger K.M., Martin N.I., van Kasteren S.I. (2021). Metabolic labeling probes for interrogation of the host-pathogen interaction. Org. Biomol. Chem..

[61] Nilsson I., Lee S.Y., Sawyer W.S., Baxter Rath C.M., Lapointe G., Six D.A. (2020). Metabolic phospholipid labeling of intact bacteria enables a fluorescence assay that detects compromised outer membranes. J. Lipid Res..

[62] van Elsas J.D., Semenov A.V., Costa R., Trevors J.T. (2011). Survival of *Escherichia coli* in the environment: fundamental and public health aspects. ISME J..

[63] Bertani G. (1951). Studies on lysogenesis I. J. Bacteriol..

[64] Blevins M.S., Klein D.R., Brodbelt J.S. (2019). Localization of cyclopropane modifications in bacterial lipids via 213 nm ultraviolet photodissociation mass spectrometry. Anal. Chem..

[65] Grogan D.W., Cronan J.E. (1986). Characterization of *Escherichia coli* mutants completely defective in synthesis of cyclopropane fatty acids. J. Bacteriol..

[66] Grant N.A., Abdel Magid A., Franklin J., Dufour Y., Lenski R.E. (2021). Changes in cell size and shape during 50,000 generations of experimental evolution with *Escherichia coli*. J. Bacteriol..

[67] Makinoshima H., Aizawa S., Hayashi H., Miki T., Nishimura A., Ishihama A. (2003). Growth phase-coupled alterations in cell structure and function of *Escherichia coli*. J. Bacteriol..

[68] Menzel J.P., Young R.S.E., Benfield A.H., Scott J.S., Wongsomboon P., Cudlman L. (2023). Ozone-enabled fatty acid discovery reveals unexpected diversity in the human lipidome. Nat. Commun..

[69] Zhao J., Fang M., Xia Y. (2021). A liquid chromatography-mass spectrometry workflow for in-depth quantitation of fatty acid double bond location isomers. J. Lipid Res..

[70] Forde B.M., Ben Zakour N.L., Stanton-Cook M., Phan M.D., Totsika M., Peters K.M. (2014). The complete genome sequence of *Escherichia coli* EC958: a high quality reference sequence for the globally disseminated multidrug resistant *E. coli* O25b:H4-ST131 clone. PLoS One.

[71] Bligh E.G., Dyer W.J. (1959). A rapid method of total lipid extraction and purification. Can. J. Biochem. Physiol..

[72] Kopczynski D., Ejsing C.S., McDonald J.G., Bamba T., Baker E.S., Bertrand-Michel J. (2024). The lipidomics reporting checklist a framework for transparency of lipidomic experiments and repurposing resource data. J. Lipid Res..

[73] Tsugawa H., Cajka T., Kind T., Ma Y., Higgins B., Ikeda K. (2015). MS-DIAL: data-independent MS/MS deconvolution for comprehensive metabolome analysis. Nat. Methods.

[74] Wickham H. (2016).

[75] Kassambara A. (2023).

[76] Dowhan W. (1992). Phosphatidylserine synthase from *Escherichia coli*. Methods Enzymol..

[77] Folch J., Lees M., Sloane Stanley G.H. (1957). A simple method for the isolation and purification of total lipides from animal tissues. J. Biol. Chem..

[78] Carman G.M., Dowhan W. (1978). A spectrophotometric method for the assay of cytidine 5'-diphospho-1,2-diacyl-*sn*-glycerol-dependent enzymes of phospholipid metabolism. J. Lipid Res..

[79] Carman G.M., Dowhan W. (1979). Phosphatidylserine synthase from *Escherichia coli*. The role of Triton X-100 in catalysis. J. Biol. Chem..

