# Supplementary material for: Endogenous formation of phosphatidylhomoserine in Escherichia coli through phosphatidylserine synthase
Source: J Biol Chem. 2025 May 20;301(7):110255. doi: 10.1016/j.jbc.2025.110255 (PMC12221286; doi:10.1016/j.jbc.2025.110255)
Supplement: Supporting information [file mmc1.pdf]

Supporting Information

Endogenous formation of phosphatidylhomoserine in *Escherichia coli* through phosphatidylserine synthase

Elise Zi Qi Ng[1], Eunju Lee[2], Shu-Sin Chng[3], Jungwook Kim[2], Xue Li Guan[1,#]

[1]Lee Kong Chian School of Medicine, Nanyang Technological University, Singapore

[2]Department of Chemistry, Gwangju Institute of Science and Technology, Gwangju, Republic of Korea.

[3]National University of Singapore, Department of Chemistry. Singapore.

[#]Corresponding author (email: xueli.guan@ntu.edu.sg)

List of content:

Supplemental Figure 1: Viability of *E. coli* BW25113 grown in M9 minimal media supplemented with 0.1% (w/v) of substrates, L-threonine, L-*allo*-threonine and L-homoserine, at 8-hour and 24-hour, visualized with colony formation on LB agar. Each condition includes two culture replicates with three technical replicates for spotting on LB agar, and is a representative set of data out of three biological replicates, (*n* = 3). Growth restrictions of *E. coli* were observed when treated with L-*allo*-threonine and L-homoserine. Nonetheless, the cells were viable and sufficient biomass was obtained for subsequent lipid analyses. Abbreviations: aThr: L-*allo*-threonine, Hse: L-homoserine, LB: Luria-Bertani broth (Miller), Thr: L-threonine

Supplemental Figure 2: Metabolic substrate incorporation in *P. aeruginosa* PAO1 cultured in M9 minimal media. (A) Viability of *P. aeruginosa* PAO1, grown in M9 minimal media supplemented with 0.1% (w/v) of substrates, L-threonine, L-*allo*-threonine and L-homoserine. For bacteria viability assessment, each experimental condition had two culture replicates, which were spotted three times technically. A total of three biological replicates were performed, (*n* = 3). Images of the spots are representative of technical replicates in one of the biological replicates. OD_{600} values were derived from the equivalent biological replicate out of three biological replicates. Left: OD_{600} (Grey dashed line: OD_{600} of blank media control). Right: colony formation. (B) XIC of three major unknown ions detected in *E. coli* (control), which was used to extract the LC/MS data obtained from *P. aeruginosa* (control) and *P. aeruginosa*, treated with L-threonine. *P. aeruginosa* when treated with L-threonine accumulated an additional peak which eluted later than the *E. coli* endogenous lipid, but earlier than the PS 34:1 synthetic standard. This supported *E. coli* did not incorporate L-threonine to form phosphatidylthreonine (PT) endogenously. XIC data was derived from one sample, and is representative of three biological replicates. Abbreviations: aThr: L-*allo*-threonine, Hse: L-homoserine, LC/MS: liquid chromatography-mass spectrometry, OD_{600}: optical density at wavelength 600 nm, PS: phosphatidylserine, Thr: L-threonine, XIC: extracted ion chromatogram

Supplemental Figure 3: Incorporation of L-homoserine at different concentrations in *E. coli* BW25113 in M9 minimal media. (A) Growth kinetics of *E. coli* BW25113 cultured in M9 minimal media supplemented with different concentrations of L-homoserine, (*n* = 3). Error bars represent standard deviation. All sampling in this work was performed at 8-hour and 24-hour, marked by the vertical dashed lines (light blue: 8-hour, dark blue: 24-hour). (B) Relative levels of PHS in *E. coli* BW25113, with increasing levels of L-homoserine introduced exogenously. Data represented the average values of two cultures for each condition. Error bars represent standard deviation. Abbreviations: Hse: L-homoserine, M9: M9 minimal media, OD_{600}: optical density at wavelength 600 nm, PHS: phosphatidylhomoserine w/v: weight per volume

Supplemental Figure 4: Growth kinetics of *E. coli* BW25113 cultured in LB and M9 minimal media supplemented with and without 0.1% (w/v) L-homoserine, ($n \geq 4$). Error bars represent standard deviation. All sampling in this work was performed at 8-hour and 24-hour, marked by the dashed lines (light blue: 8-hour, dark blue: 24-hour). *E. coli* BW25113 was able to grow better in LB-based media, compared growth in M9 minimal media. Abbreviations: Hse: L-homoserine, LB: Luria-Bertani broth (Miller), M9: M9 minimal media, OD_{600}: optical density at wavelength 600 nm

Supplemental Table 1A: List of ions in *E. coli* BW25113 at baseline conditions, characterized using negative electrospray ionization LC/MS^2. Abbreviations: CL: cardiolipin, LC/MS^2: liquid chromatography-tandem mass spectrometry, LPE: lysophosphatidylethanolamine, *m/z*: mass-to-charge ratio, PA: phosphatidic acid, PE: phosphatidylethanolamine, PG: phosphatidylglycerol, PHS: phosphatidylhomoserine

Supplemental Table 1B: Relative distribution of fatty acyl chain length (sum total of both acyl chains) within each phospholipid class [PE, PG, PHS]. Mean and standard deviation derived from three biological replicates. Abbreviations: CV: coefficient of variance, LB: Luria-Bertani broth (Miller), M9: M9 minimal media, PE: phosphatidylethanolamine, PG: phosphatidylglycerol, PHS: phosphatidylhomoserine

Supplemental Table 2: Peak areas of phospholipid classes detected in *E. coli* BW25113, cultured in LB media with, and without L-homoserine (0.1%, w/v). Abbreviations: CL: cardiolipin, Hse: L-homoserine, LB: Luria-Bertani broth (Miller), LPE: lysophosphatidylethanolamine, NA: not applicable, PA: phosphatidic acid, PE: phosphatidylethanolamine, PG: phosphatidylglycerol, PHS: phosphatidylhomoserine, PPA: phosphatidylpropanolamine

Supplemental Material: Lipidomics Minimal Reporting Checklist

Supplemental Figure 1

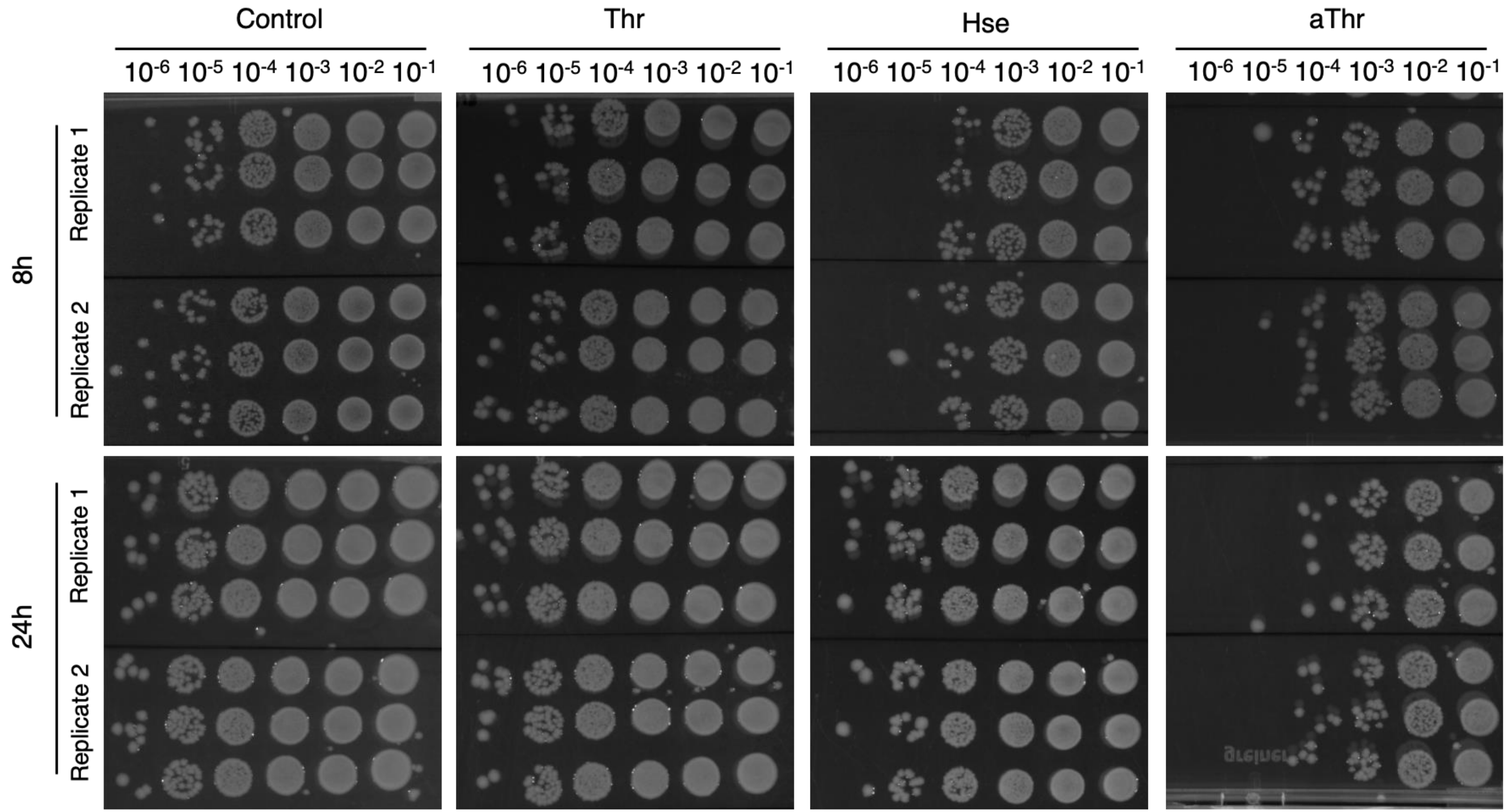

Supplemental Figure 1: Viability of *E. coli* BW25113 grown in M9 minimal media supplemented with 0.1% (w/v) of substrates, L-threonine, L-*allo*-threonine and L-homoserine, at 8-hour and 24-hour, visualized with colony formation on LB agar. Each condition includes two culture replicates with three technical replicates for spotting on LB agar, and is a representative set of data out of three biological replicates, (n = 3). Growth restrictions of *E. coli* were observed when treated with L-*allo*-threonine and L-homoserine. Nonetheless, the cells were viable and sufficient biomass was obtained for subsequent lipid analyses. Abbreviations: aThr: L-*allo*-threonine, Hse: L-homoserine, LB: Luria-Bertani broth (Miller), Thr: L-threonine

Supplemental Figure 2

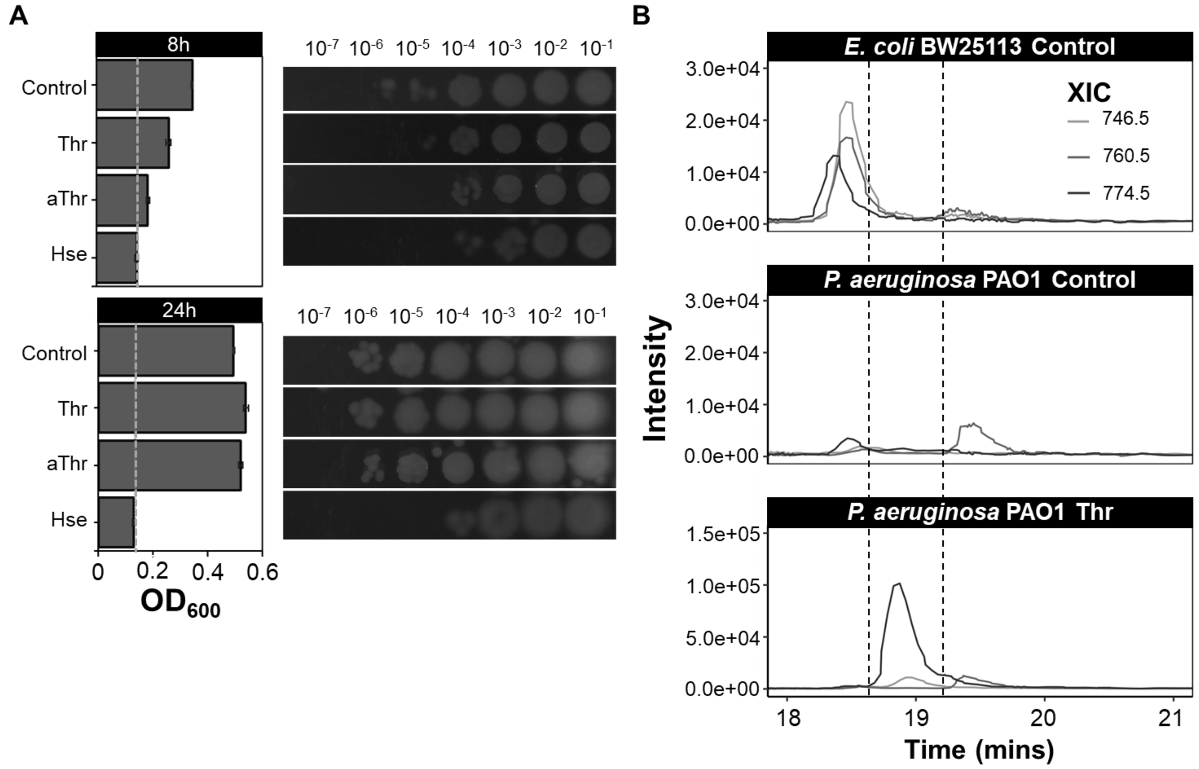

Supplemental Figure 2: Metabolic substrate incorporation in *P. aeruginosa* PAO1 cultured in M9 minimal media. (A) Viability of *P. aeruginosa* PAO1, grown in M9 minimal media supplemented with 0.1% (w/v) of substrates, L-threonine, L-*allo*-threonine and L-homoserine. For bacteria viability assessment, each experimental condition had two culture replicates, which were spotted three times technically. A total of three biological replicates were performed, (*n* = 3). Images of the spots are representative of technical replicates in one of the biological replicates. OD_{600} values were derived from the equivalent biological replicate out of three biological replicates. Left: OD_{600} (Grey dashed line: OD_{600} of blank media control). Right: colony formation. (B) XIC of three major unknown ions detected in *E. coli* (control), which was used to extract the LC/MS data obtained from *P. aeruginosa* (control) and *P. aeruginosa*, treated with L-threonine. *P. aeruginosa* when treated with L-threonine accumulated an additional peak which eluted later than the *E. coli* endogenous lipid, but earlier than the PS 34:1 synthetic standard. This supported *E. coli* did not incorporate L-threonine to form phosphatidylthreonine (PT) endogenously. XIC data was derived from one sample, and is representative of three biological replicates. Abbreviations: aThr: L-*allo*-threonine, Hse: L-homoserine, LC/MS: liquid chromatography-mass spectrometry, OD_{600}: optical density at wavelength 600 nm, PS: phosphatidylserine, Thr: L-threonine, XIC: extracted ion chromatogram

Supplemental Figure 3

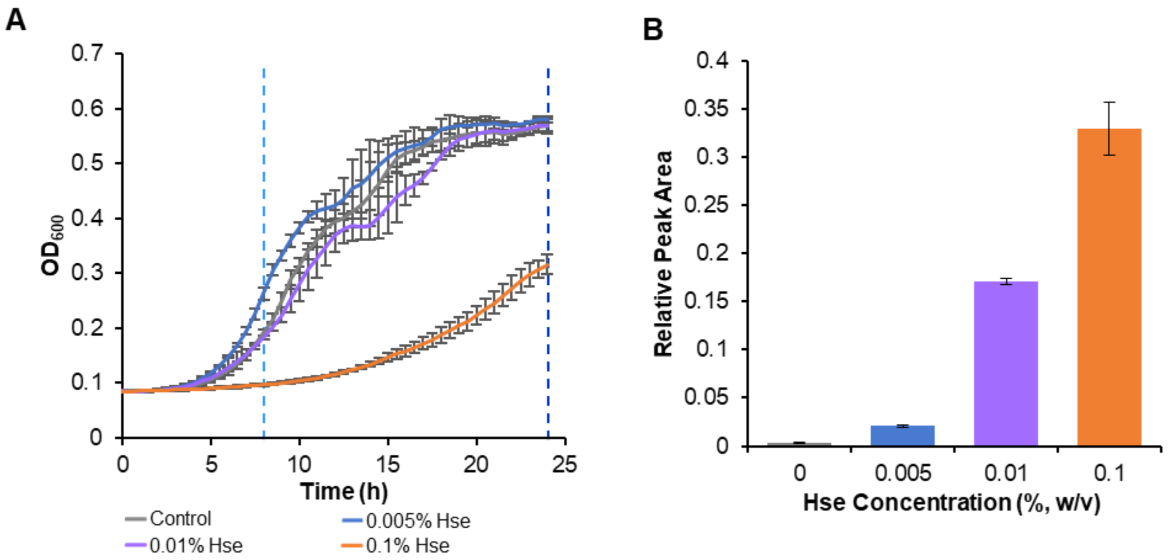

Supplemental Figure 3: Incorporation of L-homoserine at different concentrations in *E. coli* BW25113 in M9 minimal media. (A) Growth kinetics of *E. coli* BW25113 cultured in M9 minimal media supplemented with different concentrations of L-homoserine, (n = 3). Error bars represent standard deviation. All sampling in this work was performed at 8-hour and 24-hour, marked by the vertical dashed lines (light blue: 8-hour, dark blue: 24-hour). (B) Relative levels of PHS in *E. coli* BW25113, with increasing levels of L-homoserine introduced exogenously. Data represented the average values of two cultures for each condition. Error bars represent standard deviation. Abbreviations: Hse: L-homoserine, M9: M9 minimal media, OD_{600}: optical density at wavelength 600 nm, PHS: phosphatidylhomoserine w/v: weight per volume

Supplemental Figure 4

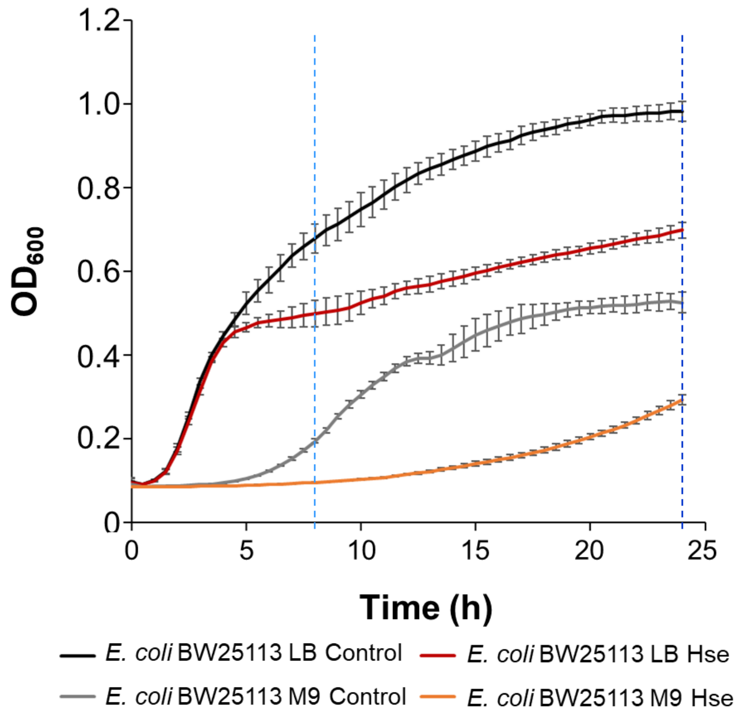

Supplemental Figure 4: Growth kinetics of *E. coli* BW25113 cultured in LB and M9 minimal media supplemented with and without 0.1% (w/v) L-homoserine, ($n \geq 4$). Error bars represent standard deviation. All sampling in this work was performed at 8-hour and 24-hour, marked by the dashed lines (light blue: 8-hour, dark blue: 24-hour). *E. coli* BW25113 was able to grow better in LB-based media, compared growth in M9 minimal media. Abbreviations: Hse: L-homoserine, LB: Luria-Bertani broth (Miller), M9: M9 minimal media, OD_{600}: optical density at wavelength 600 nm

Supplemental Table 1A: List of ions in *E. coli* BW25113 at baseline conditions, characterized using negative electrospray ionization LC/MS^2. Abbreviations: CL: cardiolipin, LC/MS^2: liquid chromatography-tandem mass spectrometry, LPE: lysophosphatidylethanolamine, *m/z*: mass-to-charge ratio, PA: phosphatidic acid, PE: phosphatidylethanolamine, PG: phosphatidylglycerol, PHS: phosphatidylhomoserine

Phospholipid class	Total fatty acyl carbon chain length	Potential maximum number of double bonds (Sum of all fatty acyls)	Average *m/z* between 3 biological replicates
CL	62	1	660.455
CL	62	2	659.450
CL	63	1	667.463
CL	63	2	666.455
CL	64	1	674.470
CL	64	2	673.467
CL	65	1	681.473
CL	65	2	680.470
CL	66	2	687.479
CL	66	3	686.470
CL	67	2	694.489
CL	68	2	701.495
CL	68	3	700.493
CL	68	4	699.479
CL	70	2	715.511
CL	70	3	714.506
CL	71	3	721.508
CL	72	3	728.518
CL	73	3	735.526
PA	32	1	645.452
PA	34	2	671.464
PA	36	2	699.496
PE	26	0	606.412
PE	27	0	620.428
PE	28	0	634.446
PE	28	1	632.430
PE	29	0	648.461
PE	29	1	646.445
PE	30	0	662.478
PE	30	1	660.460
PE	31	0	676.491
PE	31	1	674.476
PE	32	0	690.497
PE	32	1	688.492
PE	32	2	686.474
PE	33	1	702.510
PE	33	2	700.491
PE	34	0	718.503

PE	34	1	716.526
PE	34	2	714.509
PE	35	1	730.539
PE	35	2	728.522
PE	36	1	744.562
PE	36	2	742.542
PE	37	1	758.567
PE	37	2	756.552
PE	38	2	770.570
PG	28	0	665.440
PG	29	0	679.455
PG	30	0	693.472
PG	30	1	691.453
PG	31	0	707.486
PG	31	1	705.470
PG	32	0	721.489
PG	32	1	719.487
PG	32	2	717.469
PG	33	1	733.503
PG	33	2	731.484
PG	34	1	747.518
PG	34	2	745.503
PG	35	1	761.534
PG	35	2	759.516
PG	36	1	775.549
PG	36	2	773.535
PG	37	1	789.564
PG	37	2	787.550
PG	38	2	801.562
PHS	31	1	732.486
PHS	32	1	746.499
PHS	33	1	760.513
PHS	34	1	774.529
PHS	34	2	772.515
PHS	35	1	788.541
PHS	35	2	786.529
PHS	36	2	800.544
PPA	33	1	716.524
LPE	16	0	452.280
LPE	16	1	450.263
LPE	17	1	464.279
LPE	18	1	478.294

Supplemental Table 1B: Relative distribution of fatty acyl chain length (sum total of both acyl chains) within each phospholipid class [PE, PG, PHS]. Mean and standard deviation derived from three biological replicates. Abbreviations: CV: coefficient of variance, LB: Luria-Bertani broth (Miller), M9: M9 minimal media, PE: phosphatidylethanolamine, PG: phosphatidylglycerol, PHS: phosphatidylhomoserine

Species strain	Culture media	Time (h)	Lipid class	Sum fatty acyl carbon	Mean (Relative peak area within class)	Standard deviation	CV (%)
E. coli BW25113	LB	8	PE	26	9.95E-04	2.55E-04	2.56E+01
E. coli BW25113	LB	24	PE	26	5.13E-04	1.46E-04	2.86E+01
E. coli BW25113	M9	8	PE	26	2.51E-05	2.44E-05	9.71E+01
E. coli BW25113	M9	24	PE	26	1.81E-05	1.58E-05	8.72E+01
E. coli BW25113	LB	8	PE	27	1.63E-03	4.34E-04	2.66E+01
E. coli BW25113	LB	24	PE	27	1.19E-03	3.08E-04	2.59E+01
E. coli BW25113	M9	8	PE	27	1.68E-04	7.59E-05	4.53E+01
E. coli BW25113	M9	24	PE	27	1.73E-05	2.20E-05	1.27E+02
E. coli BW25113	LB	8	PE	28	1.68E-02	5.37E-03	3.19E+01
E. coli BW25113	LB	24	PE	28	1.33E-02	4.48E-03	3.37E+01
E. coli BW25113	M9	8	PE	28	3.40E-03	1.19E-03	3.49E+01
E. coli BW25113	M9	24	PE	28	4.39E-03	1.64E-03	3.73E+01
E. coli BW25113	LB	8	PE	29	2.35E-02	8.00E-03	3.40E+01
E. coli BW25113	LB	24	PE	29	1.80E-02	6.42E-03	3.58E+01
E. coli BW25113	M9	8	PE	29	7.06E-03	2.92E-03	4.13E+01
E. coli BW25113	M9	24	PE	29	3.68E-03	1.76E-03	4.77E+01
E. coli BW25113	LB	8	PE	30	8.63E-02	2.73E-02	3.17E+01
E. coli BW25113	LB	24	PE	30	8.24E-02	2.84E-02	3.44E+01
E. coli BW25113	M9	8	PE	30	5.29E-02	1.77E-02	3.34E+01
E. coli BW25113	M9	24	PE	30	7.15E-02	2.88E-02	4.02E+01
E. coli BW25113	LB	8	PE	31	6.90E-02	1.53E-02	2.22E+01
E. coli BW25113	LB	24	PE	31	6.84E-02	1.55E-02	2.27E+01
E. coli BW25113	M9	8	PE	31	5.42E-02	6.60E-03	1.22E+01
E. coli BW25113	M9	24	PE	31	4.72E-02	1.50E-02	3.19E+01
E. coli BW25113	LB	8	PE	32	1.23E-01	5.07E-03	4.11E+00
E. coli BW25113	LB	24	PE	32	1.64E-01	4.69E-03	2.86E+00
E. coli BW25113	M9	8	PE	32	2.74E-01	1.15E-02	4.20E+00
E. coli BW25113	M9	24	PE	32	6.07E-02	4.79E-03	7.90E+00
E. coli BW25113	LB	8	PE	33	2.95E-01	3.85E-02	1.31E+01
E. coli BW25113	LB	24	PE	33	2.88E-01	3.69E-02	1.28E+01
E. coli BW25113	M9	8	PE	33	1.51E-01	1.00E-02	6.63E+00
E. coli BW25113	M9	24	PE	33	4.95E-01	4.51E-02	9.11E+00
E. coli BW25113	LB	8	PE	34	1.18E-01	2.26E-02	1.92E+01
E. coli BW25113	LB	24	PE	34	1.51E-01	1.86E-02	1.23E+01
E. coli BW25113	M9	8	PE	34	2.84E-01	2.09E-02	7.38E+00
E. coli BW25113	M9	24	PE	34	4.71E-02	7.74E-03	1.64E+01
E. coli BW25113	LB	8	PE	35	1.37E-01	1.32E-02	9.67E+00
E. coli BW25113	LB	24	PE	35	1.10E-01	9.49E-03	8.62E+00
E. coli BW25113	M9	8	PE	35	5.87E-02	2.10E-03	3.58E+00
E. coli BW25113	M9	24	PE	35	1.45E-01	9.90E-03	6.84E+00
E. coli BW25113	LB	8	PE	36	7.71E-02	5.36E-03	6.95E+00
E. coli BW25113	LB	24	PE	36	7.20E-02	6.99E-03	9.71E+00
E. coli BW25113	M9	8	PE	36	1.09E-01	1.34E-02	1.23E+01
E. coli BW25113	M9	24	PE	36	8.32E-02	2.57E-03	3.09E+00
E. coli BW25113	LB	8	PE	37	3.91E-02	1.10E-02	2.82E+01

E. coli BW25113	LB	24	PE	37	1.91E-02	3.56E-03	1.86E+01
E. coli BW25113	M9	8	PE	37	4.71E-03	6.52E-04	1.39E+01
E. coli BW25113	M9	24	PE	37	9.76E-03	2.61E-03	2.67E+01
E. coli BW25113	LB	8	PE	38	1.25E-02	5.46E-03	4.38E+01
E. coli BW25113	LB	24	PE	38	1.25E-02	3.56E-03	2.86E+01
E. coli BW25113	M9	8	PE	38	7.57E-04	3.57E-04	4.72E+01
E. coli BW25113	M9	24	PE	38	3.23E-02	9.98E-03	3.09E+01
E. coli BW25113	LB	8	PG	28	4.32E-03	5.59E-04	1.29E+01
E. coli BW25113	LB	24	PG	28	2.34E-03	3.56E-04	1.52E+01
E. coli BW25113	M9	8	PG	28	1.22E-04	2.29E-05	1.87E+01
E. coli BW25113	M9	24	PG	28	7.59E-04	1.50E-04	1.98E+01
E. coli BW25113	LB	8	PG	29	5.20E-03	8.00E-04	1.54E+01
E. coli BW25113	LB	24	PG	29	3.32E-03	6.90E-04	2.08E+01
E. coli BW25113	M9	8	PG	29	3.89E-04	1.92E-05	4.94E+00
E. coli BW25113	M9	24	PG	29	1.62E-04	4.85E-05	2.99E+01
E. coli BW25113	LB	8	PG	30	3.34E-02	3.42E-03	1.02E+01
E. coli BW25113	LB	24	PG	30	2.69E-02	3.37E-03	1.25E+01
E. coli BW25113	M9	8	PG	30	1.07E-02	9.84E-04	9.16E+00
E. coli BW25113	M9	24	PG	30	1.41E-02	1.63E-03	1.16E+01
E. coli BW25113	LB	8	PG	31	3.82E-02	1.79E-03	4.69E+00
E. coli BW25113	LB	24	PG	31	4.29E-02	3.26E-03	7.58E+00
E. coli BW25113	M9	8	PG	31	1.83E-02	1.35E-03	7.38E+00
E. coli BW25113	M9	24	PG	31	2.50E-02	1.02E-03	4.06E+00
E. coli BW25113	LB	8	PG	32	1.19E-01	5.05E-03	4.25E+00
E. coli BW25113	LB	24	PG	32	2.02E-01	7.82E-04	3.88E-01
E. coli BW25113	M9	8	PG	32	2.51E-01	7.31E-03	2.91E+00
E. coli BW25113	M9	24	PG	32	3.35E-02	3.58E-03	1.07E+01
E. coli BW25113	LB	8	PG	33	2.90E-01	8.15E-03	2.81E+00
E. coli BW25113	LB	24	PG	33	2.98E-01	3.93E-03	1.32E+00
E. coli BW25113	M9	8	PG	33	9.80E-02	2.56E-03	2.62E+00
E. coli BW25113	M9	24	PG	33	5.33E-01	4.66E-03	8.74E-01
E. coli BW25113	LB	8	PG	34	1.51E-01	7.19E-03	4.77E+00
E. coli BW25113	LB	24	PG	34	1.95E-01	2.89E-03	1.48E+00
E. coli BW25113	M9	8	PG	34	3.73E-01	2.14E-03	5.74E-01
E. coli BW25113	M9	24	PG	34	3.00E-02	2.40E-03	8.02E+00
E. coli BW25113	LB	8	PG	35	2.12E-01	4.43E-03	2.09E+00
E. coli BW25113	LB	24	PG	35	1.42E-01	6.89E-03	4.85E+00
E. coli BW25113	M9	8	PG	35	5.45E-02	1.26E-03	2.31E+00
E. coli BW25113	M9	24	PG	35	2.59E-01	3.54E-03	1.37E+00
E. coli BW25113	LB	8	PG	36	9.01E-02	2.86E-03	3.17E+00
E. coli BW25113	LB	24	PG	36	6.13E-02	9.63E-04	1.57E+00
E. coli BW25113	M9	8	PG	36	1.88E-01	1.12E-02	5.95E+00
E. coli BW25113	M9	24	PG	36	6.61E-02	3.68E-03	5.56E+00
E. coli BW25113	LB	8	PG	37	4.17E-02	2.77E-03	6.65E+00
E. coli BW25113	LB	24	PG	37	1.48E-02	1.33E-03	8.97E+00
E. coli BW25113	M9	8	PG	37	5.48E-03	1.23E-04	2.26E+00
E. coli BW25113	M9	24	PG	37	7.30E-03	8.08E-04	1.11E+01
E. coli BW25113	LB	8	PG	38	1.51E-02	2.80E-03	1.86E+01
E. coli BW25113	LB	24	PG	38	1.17E-02	1.40E-03	1.20E+01
E. coli BW25113	M9	8	PG	38	7.94E-04	1.98E-04	2.49E+01
E. coli BW25113	M9	24	PG	38	3.07E-02	3.01E-03	9.81E+00
E. coli BW25113	LB	8	PHS	31	4.54E-02	9.43E-03	2.08E+01
E. coli BW25113	LB	24	PHS	31	4.83E-02	7.40E-03	1.53E+01
E. coli BW25113	M9	8	PHS	31	5.60E-02	1.25E-02	2.23E+01
E. coli BW25113	M9	24	PHS	31	1.16E-01	1.08E-01	9.28E+01
E. coli BW25113	LB	8	PHS	32	1.21E-01	6.65E-03	5.49E+00

E. coli BW25113	LB	24	PHS	32	1.94E-01	4.21E-02	2.17E+01
E. coli BW25113	M9	8	PHS	32	2.45E-01	1.36E-02	5.55E+00
E. coli BW25113	M9	24	PHS	32	2.67E-02	3.08E-02	1.16E+02
E. coli BW25113	LB	8	PHS	33	4.33E-01	1.97E-02	4.55E+00
E. coli BW25113	LB	24	PHS	33	3.90E-01	2.85E-02	7.30E+00
E. coli BW25113	M9	8	PHS	33	1.60E-01	9.64E-03	6.03E+00
E. coli BW25113	M9	24	PHS	33	5.41E-01	3.17E-02	5.86E+00
E. coli BW25113	LB	8	PHS	34	1.80E-01	1.40E-02	7.81E+00
E. coli BW25113	LB	24	PHS	34	2.03E-01	1.84E-02	9.05E+00
E. coli BW25113	M9	8	PHS	34	3.85E-01	2.08E-02	5.40E+00
E. coli BW25113	M9	24	PHS	34	1.41E-01	6.59E-02	4.68E+01
E. coli BW25113	LB	8	PHS	35	1.63E-01	1.65E-02	1.01E+01
E. coli BW25113	LB	24	PHS	35	1.16E-01	2.04E-02	1.76E+01
E. coli BW25113	M9	8	PHS	35	8.24E-02	1.90E-02	2.31E+01
E. coli BW25113	M9	24	PHS	35	1.41E-01	2.00E-02	1.41E+01
E. coli BW25113	LB	8	PHS	36	5.75E-02	6.31E-03	1.10E+01
E. coli BW25113	LB	24	PHS	36	4.79E-02	6.22E-03	1.30E+01
E. coli BW25113	M9	8	PHS	36	7.16E-02	1.48E-02	2.07E+01
E. coli BW25113	M9	24	PHS	36	3.37E-02	2.54E-02	7.55E+01

Supplemental Table 2: Peak areas of phospholipid classes detected in *E. coli* BW25113, cultured in LB media with, and without $_{L}$-homoserine (0.1%, w/v). Abbreviations: CL: cardiolipin, Hse: $_{L}$-homoserine, LB: Luria-Bertani broth (Miller), LPE: lysophosphatidylethanolamine, NA: not applicable, PA: phosphatidic acid, PE: phosphatidylethanolamine, PG: phosphatidylglycerol, PHS: phosphatidylhomoserine, PPA: phosphatidylpropanolamine

**Biological replicate**	**Sample/Species strain**	**Media**	**Substrate**	**Time (h)**	**CL**	**PA**	**PE**	**PG**	**PHS**	**PPA**	**LPE**
1	Mobile phase blank	NA	NA	NA	1.922E+06	3.510E+05	1.950E+06	1.300E+06	1.078E+06	1.127E+06	1.162E+06
1	*E. coli* BW25113	LB	Control	8	9.288E+06	5.161E+05	1.129E+08	1.949E+08	2.193E+06	2.678E+06	1.530E+06
1	*E. coli* BW25113	LB	Hse	8	1.467E+07	5.174E+05	6.389E+07	1.069E+07	2.161E+08	3.439E+07	1.288E+06
1	*E. coli* BW25113	LB	Control	24	3.400E+07	6.698E+05	1.594E+08	1.928E+08	2.672E+06	3.977E+06	2.487E+06
1	*E. coli* BW25113	LB	Hse	24	2.272E+07	5.825E+05	6.526E+07	3.304E+07	1.795E+08	5.813E+07	1.263E+06
2	Mobile phase blank	NA	NA	NA	1.084E+06	4.162E+05	1.359E+06	1.181E+06	6.572E+05	8.265E+05	3.937E+05
2	*E. coli* BW25113	LB	Control	8	7.218E+06	5.701E+05	1.049E+08	1.820E+08	2.271E+06	2.128E+06	1.490E+06
2	*E. coli* BW25113	LB	Hse	8	9.559E+06	5.546E+05	5.310E+07	1.031E+07	2.185E+08	2.402E+07	9.459E+05
2	*E. coli* BW25113	LB	Control	24	1.989E+07	5.336E+05	1.039E+08	1.316E+08	1.493E+06	2.187E+06	1.687E+06
2	*E. coli* BW25113	LB	Hse	24	1.264E+07	6.033E+05	4.985E+07	2.397E+07	1.766E+08	4.215E+07	1.028E+06
3	Mobile phase blank	NA	NA	NA	1.234E+06	7.888E+05	1.227E+06	9.618E+05	8.892E+05	6.035E+05	3.730E+05
3	*E. coli* BW25113	LB	Control	8	2.015E+07	1.240E+06	1.087E+08	2.091E+08	4.515E+06	3.213E+06	1.635E+06
3	*E. coli* BW25113	LB	Hse	8	2.596E+07	9.262E+05	7.590E+07	1.471E+07	2.523E+08	5.182E+07	9.422E+05
3	*E. coli* BW25113	LB	Control	24	4.567E+07	9.948E+05	1.128E+08	1.537E+08	2.843E+06	4.321E+06	2.263E+06
3	*E. coli* BW25113	LB	Hse	24	3.930E+07	1.101E+06	7.209E+07	3.878E+07	2.353E+08	7.093E+07	1.110E+06

Contents of Report

Created by https://lipidomicstandards.org, version v2.4.0

Separation Workflow 1
Overall study design . . . 1
Lipid extraction . . . 1
Analytical platform . . . 2
Quality control . . . 2
Method qualification and validation . . . 2
Reporting . . . 2

Sample Descriptions 3
E. coli BW25113 / Bacteria / Cells . . . 3

Lipid Class Descriptions 3
1) LPE[M-H]- / Lipid identification . . . 3
1) LPE[M-H]- / Lipid quantification . . . 4
2) CL[M-2H]2- / Lipid identification . . . 4
2) CL[M-2H]2- / Lipid quantification . . . 4
3) PE[M-H]- / Lipid identification . . . 5
3) PE[M-H]- / Lipid quantification . . . 5
4) PG[M-H]- / Lipid identification . . . 6
4) PG[M-H]- / Lipid quantification . . . 6
5) PA[M-H]- / Lipid identification . . . 7
5) PA[M-H]- / Lipid quantification . . . 7
6) LPG[M-H]- / Lipid identification . . . 8
6) LPG[M-H]- / Lipid quantification . . . 8
7) Phosphatidylhomoserine[M-H]- / Lipid identification . . . 9
7) Phosphatidylhomoserine[M-H]- / Lipid quantification . . . 9
8) Phosphatidylpropanolamine[M-H]- / Lipid identification . . . 10
8) Phosphatidylpropanolamine[M-H]- / Lipid quantification . . . 10
9) Acyl phosphatidylglycerol[M-H]- / Lipid identification . . . 11
9) Acyl phosphatidylglycerol[M-H]- / Lipid quantification . . . 11

Separation Workflow

Overall study design

Title of the study	Revisiting Lipidome of E. coli		
Document creation date	08/04/2024	Corresponding Email	xueli.guan@gmail.com
Principal investigator	Guan Xue Li	Is the workflow targeted or untargeted?	Untargeted
Institution	Nanyang Technological University	Clinical	No

Lipid extraction

Extraction method	2-phase system	Were internal standards added prior extraction?	Yes
pH adjustment	None	Special conditions	-
2-phase system	Bligh&Dyer	Derivatization	-

Analytical platform

Ionizaton additives	Ammonia	MS Level	MS2
Number of separation dimensions	One dimension	Mass window for precursor ion isolation (in Da total isolation window)	1
Separation type 1	LC	Mass resolution for detected ion at MS2	High resolution
Separation mode 1 (liquid)	NP	Resolution at m/z 200 at MS2	25000
Detector	Mass spectrometer	Mass accuracy in ppm at MS2	10
MS type	QTOF	Recording mode of raw data at MS2	Profile mode
MS vendor	SCIEX	Was/Were additional dimension/techniques used	No
Ion source	ESI		

Quality control

Blanks	Yes	Quality control	No
Type of Blanks	Extraction blank, Solvent blank		

Method qualification and validation

Method validation	Yes	Precision	No
Lipid recovery	No	Accuracy	No
Dynamic quantification range	No	Guidelines followed	None
Limit of quantitation (LOQ)/Limit of detection (LOD)	No		

Reporting

Are reported raw data uploaded into repository?	Available on request	Raw data upload	No
Are metadata available?	No	Additional comments	-

Sample Descriptions

E. coli BW25113 / Bacteria / Cells

Storage and collection conditions	Available	Storage time (month)	2
Provided preanalytical information	Time to freeze (min), Storage time (month), Freeze-thaw cycles	Freeze-thaw cycles	0
Temperature handling original sample	4-8 °C	Additives	None
Instant sample preparation	No	Were samples stored under inert gas?	No
Time to freeze (min)	120	Additional preservation methods	No
Snap freezing in liquid N2	Yes	Biobank samples	No
Storage temperature	-80 °C		

Lipid Class Descriptions

1) LPE[M-H]- / Lipid identification

Lipid class	LPE	Limit of detection	No
MS Level for identification	MS2	RT verified by standard	Yes
Identification level	Species level	Separation of isobaric/isomeric interferece confirmed	No
Polarity mode	Negative	Model for separation prediction	No
Type of negative (precursor)ion	[M-H]-	Additional dimension/techniques	-
Fragments for identification	Fragment name: -FA1(-H); GP(153)	Lipid Identification Software	MS-DIAL
Isotope correction at MS2	No	Data manipulation	-
MS2 verified by standard	Yes	Nomenclature for intact lipid molecule	No
Background check at MS2	Yes	Nomenclature for fragment ions	N/A
Did you presume assumptions for identification?	No	Further identification remarks	-
Check on:	-		

1) LPE[M-H]- / Lipid quantification

Quantitative	No	Batch correction	No
Normalization to reference	No	Further quantification remarks	Involved comparative analysis of paired samples, based on relative proportion of peak areas. this is due to the lack of pertinent standards for this study.

2) CL[M-2H]2- / Lipid identification

Lipid class	CL	Limit of detection	No
MS Level for identification	MS2	RT verified by standard	Yes
Identification level	Undefined	Separation of isobaric/isomeric interferece confirmed	No
Polarity mode	Negative	Model for separation prediction	No
Type of negative (precursor)ion	[M-2H]2-	Additional dimension/techniques	-
Fragments for identification		Lipid Identification Software	MS-DIAL

Fragment name
-FA1(-H) [-1]
-FA2(-H) [-2]
-FA3(-H) [-1]

Isotope correction at MS2	No	Data manipulation	-
MS2 verified by standard	Yes	Nomenclature for intact lipid molecule	No
Background check at MS2	Yes	Nomenclature for fragment ions	N/A
Did you presume assumptions for identification?	No	Further identification remarks	we did not specifically report the fatty acyl composition as our analytical method does not separate fatty acid chain modification e.g cyclopropanation
Check on:	-		

2) CL[M-2H]2- / Lipid quantification

Quantitative	No	Batch correction	No
Normalization to reference	No	Further quantification remarks	Involved comparative analysis of paired samples, based on relative proportion of peak areas. this is due to the lack of pertinent standards for this study.

3) PE[M-H]- / Lipid identification

Lipid class	PE	Limit of detection	No
MS Level for identification	MS2	RT verified by standard	Yes
Identification level	Undefined	Separation of isobaric/isomeric interferece confirmed	No
Polarity mode	Negative	Model for separation prediction	No
Type of negative (precursor)ion	[M-H]-	Additional dimension/techniques	-
Fragments for identification		Lipid Identification Software	MS-DIAL

Fragment name
GP(153)
HG(PE,196)
-FA1(-H)
HG(PE,140)
-FA2(-H)

Isotope correction at MS2	No	Data manipulation	-
MS2 verified by standard	Yes	Nomenclature for intact lipid molecule	No
Background check at MS2	Yes	Nomenclature for fragment ions	N/A
Did you presume assumptions for identification?	No	Further identification remarks	we did not specifically report the fatty acyl composition as our analytical method does not separate fatty acid chain modification e.g cyclopropanation
Check on:	-		

3) PE[M-H]- / Lipid quantification

Quantitative	No	Batch correction	No
Normalization to reference	No	Further quantification remarks	Involved comparative analysis of paired samples, based on relative proportion of peak areas. this is due to the lack of pertinent standards for this study.

4) PG[M-H]- / Lipid identification

Lipid class	PG	Limit of detection	No
MS Level for identification	MS2	RT verified by standard	Yes
Identification level	Undefined	Separation of isobaric/isomeric interferece confirmed	No
Polarity mode	Negative	Model for separation prediction	No
Type of negative (precursor)ion	[M-H]-	Additional dimension/techniques	-
Fragments for identification		Lipid Identification Software	MS-DIAL

Fragment name
GP(153)
-FA1(-H)
-FA2(-H)
HG(PG,171)

Isotope correction at MS2	No	Data manipulation	-
MS2 verified by standard	Yes	Nomenclature for intact lipid molecule	No
Background check at MS2	Yes	Nomenclature for fragment ions	N/A
Did you presume assumptions for identification?	No	Further identification remarks	we did not specifically report the fatty acyl composition as our analytical method does not separate fatty acid chain modification e.g cyclopropanation
Check on:	-		

4) PG[M-H]- / Lipid quantification

Quantitative	No	Batch correction	No
Normalization to reference	No	Further quantification remarks	Involved comparative analysis of paired samples, based on relative proportion of peak areas. this is due to the lack of pertinent standards for this study.

5) PA[M-H]- / Lipid identification

Lipid class	PA	Limit of detection	No
MS Level for identification	MS2	RT verified by standard	Yes
Identification level	Undefined	Separation of isobaric/isomeric interferece confirmed	No
Polarity mode	Negative	Model for separation prediction	No
Type of negative (precursor)ion	[M-H]-	Additional dimension/techniques	-
Fragments for identification		Lipid Identification Software	MS-DIAL

Fragment name
GP(153)
-FA1(-H)
-FA2(-H)
P(79)

Isotope correction at MS2	No	Data manipulation	-
MS2 verified by standard	Yes	Nomenclature for intact lipid molecule	No
Background check at MS2	Yes	Nomenclature for fragment ions	N/A
Did you presume assumptions for identification?	No	Further identification remarks	we did not specifically report the fatty acyl composition as our analytical method does not separate fatty acid chain modification e.g cyclopropanation
Check on:	-		

5) PA[M-H]- / Lipid quantification

Quantitative	No	Batch correction	No
Normalization to reference	No	Further quantification remarks	Involved comparative analysis of paired samples, based on relative proportion of peak areas. this is due to the lack of pertinent standards for this study.

6) LPG[M-H]- / Lipid identification

Lipid class	LPG	Limit of detection	No
MS Level for identification	MS2	RT verified by standard	Yes
Identification level	Species level	Separation of isobaric/isomeric interferece confirmed	No
Polarity mode	Negative	Model for separation prediction	No
Type of negative (precursor)ion	[M-H]-	Additional dimension/techniques	-
Fragments for identification		Lipid Identification Software	MS-DIAL

Fragment name
-FA1(-H)
GP(153)
P(79)

Isotope correction at MS2	No	Data manipulation	-
MS2 verified by standard	Yes	Nomenclature for intact lipid molecule	No
Background check at MS2	Yes	Nomenclature for fragment ions	N/A
Did you presume assumptions for identification?	No	Further identification remarks	-
Check on:	-		

6) LPG[M-H]- / Lipid quantification

Quantitative	No	Batch correction	No
Normalization to reference	No	Further quantification remarks	No quantitation work was done

7) Phosphatidylhomoserine[M-H]- / Lipid identification

Lipid class	Phosphatidylhomoserine	Limit of detection	No
MS Level for identification	MS2	RT verified by standard	No
Identification level	Undefined	Separation of isobaric/isomeric interferece confirmed	No
Polarity mode	Negative	Model for separation prediction	No
Type of negative (precursor)ion	[M-H]-	Additional dimension/techniques	-
Fragments for identification	**Fragment name**<br>GP(153)<br>-FA1(-H)<br>-FA2(-H)	Lipid Identification Software	The structure was solved de novo using primary MSMS data and other supporting methods
Isotope correction at MS2	No	Data manipulation	-
MS2 verified by standard	Yes	Nomenclature for intact lipid molecule	No
Background check at MS2	Yes	Nomenclature for fragment ions	N/A
Did you presume assumptions for identification?	No	Further identification remarks	this is an uncommon class of phospholipid which we have proven using substrate incorporate. no synthetic standard is available. we did not specifically report the fatty acyl composition as our analytical method does not separate fatty acid chain modification e.g cyclopropanation
Check on:	-		

7) Phosphatidylhomoserine[M-H]- / Lipid quantification

Quantitative	No	Batch correction	No
Normalization to reference	No	Further quantification remarks	Involved comparative analysis of paired samples, based on relative proportion of peak areas. this is due to the lack of pertinent standards for this study.

8) Phosphatidylpropanolamine[M-H]- / Lipid identification

Lipid class	Phosphatidylpropanolamine	Limit of detection	No
MS Level for identification	MS2	RT verified by standard	No
Identification level	Undefined	Separation of isobaric/isomeric interferece confirmed	No
Polarity mode	Negative	Model for separation prediction	No
Type of negative (precursor)ion	[M-H]-	Additional dimension/techniques	-
Fragments for identification	**Fragment name**<br>-FA1(-H)<br>-FA2(-H)	Lipid Identification Software	The structure was solved de novo using primary MSMS data and other supporting methods
Isotope correction at MS2	No	Data manipulation	-
MS2 verified by standard	Yes	Nomenclature for intact lipid molecule	No
Background check at MS2	Yes	Nomenclature for fragment ions	N/A
Did you presume assumptions for identification?	No	Further identification remarks	this is an uncommon class of phospholipid which we have proven using substrate incorporate. no synthetic standard is available. we did not specifically report the fatty acyl composition as our analytical method does not separate fatty acid chain modification e.g cyclopropanation
Check on:	-		

8) Phosphatidylpropanolamine[M-H]- / Lipid quantification

Quantitative	No	Batch correction	No
Normalization to reference	No	Further quantification remarks	Involved comparative analysis of paired samples, based on relative proportion of peak areas. this is due to the lack of pertinent standards for this study.

9) Acyl phosphatidylglycerol[M-H]- / Lipid identification

Lipid class	Acyl phosphatidylglycerol	Limit of detection	No
MS Level for identification	MS2	RT verified by standard	Yes
Identification level	Undefined	Separation of isobaric/isomeric interferece confirmed	No
Polarity mode	Negative	Model for separation prediction	No
Type of negative (precursor)ion	[M-H]-	Additional dimension/techniques	-
Fragments for identification		Lipid Identification Software	MS-DIAL

Fragment name
-FA1(-H)
-FA2(-H)

Isotope correction at MS2	No	Data manipulation	-
MS2 verified by standard	No	Nomenclature for intact lipid molecule	No
Background check at MS2	Yes	Nomenclature for fragment ions	N/A
Did you presume assumptions for identification?	No	Further identification remarks	we did not specifically report the fatty acyl composition as our analytical method does not separate fatty acid chain modification e.g cyclopropanation
Check on:	-		

9) Acyl phosphatidylglycerol[M-H]- / Lipid quantification

Quantitative	No	Batch correction	No
Normalization to reference	No	Further quantification remarks	No quantitation work was performed
